# A higher‐yield hybrid rice is achieved by assimilating a dominant heterotic gene in inbred parental lines

**DOI:** 10.1111/pbi.14295

**Published:** 2024-03-07

**Authors:** Changsheng Wang, Ziqun Wang, Yunxiao Cai, Zhou Zhu, Danheng Yu, Lei Hong, Yongchun Wang, Wei Lv, Qiang Zhao, Lizhen Si, Kun Liu, Bin Han

**Affiliations:** ^1^ National Center for Gene Research, State Key Laboratory of Plant Molecular Genetics, Center for Excellence in Molecular Plant Sciences, Institute of Plant Physiology and Ecology Chinese Academy of Sciences Shanghai China; ^2^ School of Life Science and Technology ShanghaiTech University Shanghai China; ^3^ Department of Life Sciences, Imperial College London South Kensington London UK

**Keywords:** heterosis, crispr/cas9, rice, breeding, grian yield, grain size

## Abstract

The exploitation of heterosis to integrate parental advantages is one of the fastest and most efficient ways of rice breeding. The genomic architecture of heterosis suggests that the grain yield is strongly correlated with the accumulation of numerous rare superior alleles with positive dominance. However, the improvements in yield of hybrid rice have shown a slowdown or even plateaued due to the limited availability of complementary superior alleles. In this study, we achieved a considerable increase in grain yield of restorer lines by inducing an alternative splicing event in a heterosis gene *OsMADS1* through CRISPR‐Cas9, which accounted for approximately 34.1%–47.5% of yield advantage over their corresponding inbred rice cultivars. To achieve a higher yield in hybrid rice, we crossed the gene‐edited restorer parents harbouring *OsMADS1* ^*GW3p6*^ with the sterile lines to develop new rice hybrids. In two‐line hybrid rice Guang‐liang‐you 676 (GLY676), the yield of modified hybrids carrying the homozygous heterosis gene *OsMADS1* ^*GW3p6*^ significantly exceeded that of the original hybrids with heterozygous *OsMADS1.* Similarly, the gene‐modified F_1_ hybrids with heterozygous *OsMADS1* ^*GW3p6*^ increased grain yield by over 3.4% compared to the three‐line hybrid rice Quan‐you‐si‐miao (QYSM) with the homozygous genotype of *OsMADS1*. Our study highlighted the great potential in increasing the grain yield of hybrid rice by pyramiding a single heterosis gene via CRISPR‐Cas9. Furthermore, these results demonstrated that the incomplete dominance of heterosis genes played a major role in yield‐related heterosis and provided a promising strategy for breeding higher‐yielding rice varieties above what is currently achievable.

## Introduction

Heterosis, or hybrid vigour, describes the situation in which hybrid offspring exhibit superior phenotypic performance than that of their parents. Hybrid rice often shows a 10%–20% yield advantage over their corresponding inbred rice cultivars. The exploitation of heterosis has been widely applied in hybrid rice breeding and has achieved remarkable achievements in grain yield. Several classical hypotheses to explain heterosis were proposed soon after plant breeders began to purposefully exploit heterosis, for example, three non‐mutually exclusive hypotheses: dominance, overdominance and epistasis. Among them, dominance and overdominance are the most prominent genetic models for the genetic basis of heterosis. And extensive studies in multiple plants have provided support for these genetic models (Krieger *et al*., 2010; Liu *et al*., 2020a; Seymour *et al*., 2016; Torgeman and Zamir, 2023; Wang *et al*., 2023; Xiao *et al*., 1995; Zhou *et al*., 2012). In search of the genetic basis of heterosis, a direct approach is to evaluate the contributions of heterosis quantitative trait loci (hQTL) to the heterotic advantage through the analysis of their heterotic effects (Lippman and Zamir, 2007; Schnable and Springer, 2013). Recently, the genomic architecture of rice heterosis for yield‐related traits revealed that the grain yield was strongly correlated with the accumulation of numerous rare superior alleles with positive dominance, and a small number of heterosis loci from female parents explained a large proportion of the yield advantage of hybrids over their male parents (Huang *et al*., 2015, 2016). Although the genetic basis of rice heterosis has well been elucidated, it is still labour‐intensive and time‐consuming to exploit the heterotic effect from a crossing hybridization in crop hybrid breeding.

CRISPR‐Cas‐based genome editing is a powerful and transformative method to introduce predictable and precise genome modifications into plants to obtain desired traits, which holds great promise for crop improvement (Gao, 2021). To date, genome editing has been successfully applied to improve multiple agronomic traits in various crops, such as grain yield (Song *et al*., 2022), grain quality (Xu *et al*., 2021), and disease‐resistance traits (Wei *et al*., 2021b). Therefore, based on a better understanding of heterosis, there is still significant potential for the development of hybrid breeding improvements through gene editing technologies. Alternative splicing (AS) is pervasive to increase transcriptomic and proteomic complexity, which contributed potentially to genomic diversity to regulate complex traits (Wang and Brendel, 2006). Accumulating evidence in rice supports that AS induced by variation at either intron donor or acceptor sites could modulate important agronomic traits, such as *Waxy* controlling grain quality (Cai *et al*., 1998) and *RLI1* controlling Pi starvation signalling and growth (Guo *et al*., 2022). Recently, it was found that mutations at splice sites could cause messenger RNA (mRNA) missplicing and gene disruption through CRISPR‐Cas9 systems (Li *et al*., 2019; Tang *et al*., 2021).

In this study, we achieved an alternative 3′ splicing event of the heterosis gene *OsMADS1* in the male parent by disrupting the 3′ splice site through the CRISPR‐Cas9 system. The gene‐edited restore line with the AS of *OsMADS1* displayed a higher yield and better grain quality than wild‐type plants. Notably, the modified hybrids harbouring a homozygous or heterozygous genotype of the heterosis gene (*OsMADS1* ^*GW3p6*^) under the same hybrid genetic background have a better performance in grain yield compared to the original hybrid variety with heterozygous *OsMADS1* ^*GW3p6*^ *or* homozygous *OsMADS1*, respectively. Our results implied that the dominance effect underlying functional complementation was an indispensable contributor to yield heterosis and provided a valuable breeding target and an efficient method for hybrid rice breeding without compromising heterotic performance.

## Results

### A strategy to improve hybrid breeding based on the genetic basis of rice heterosis

**Figure 1:**
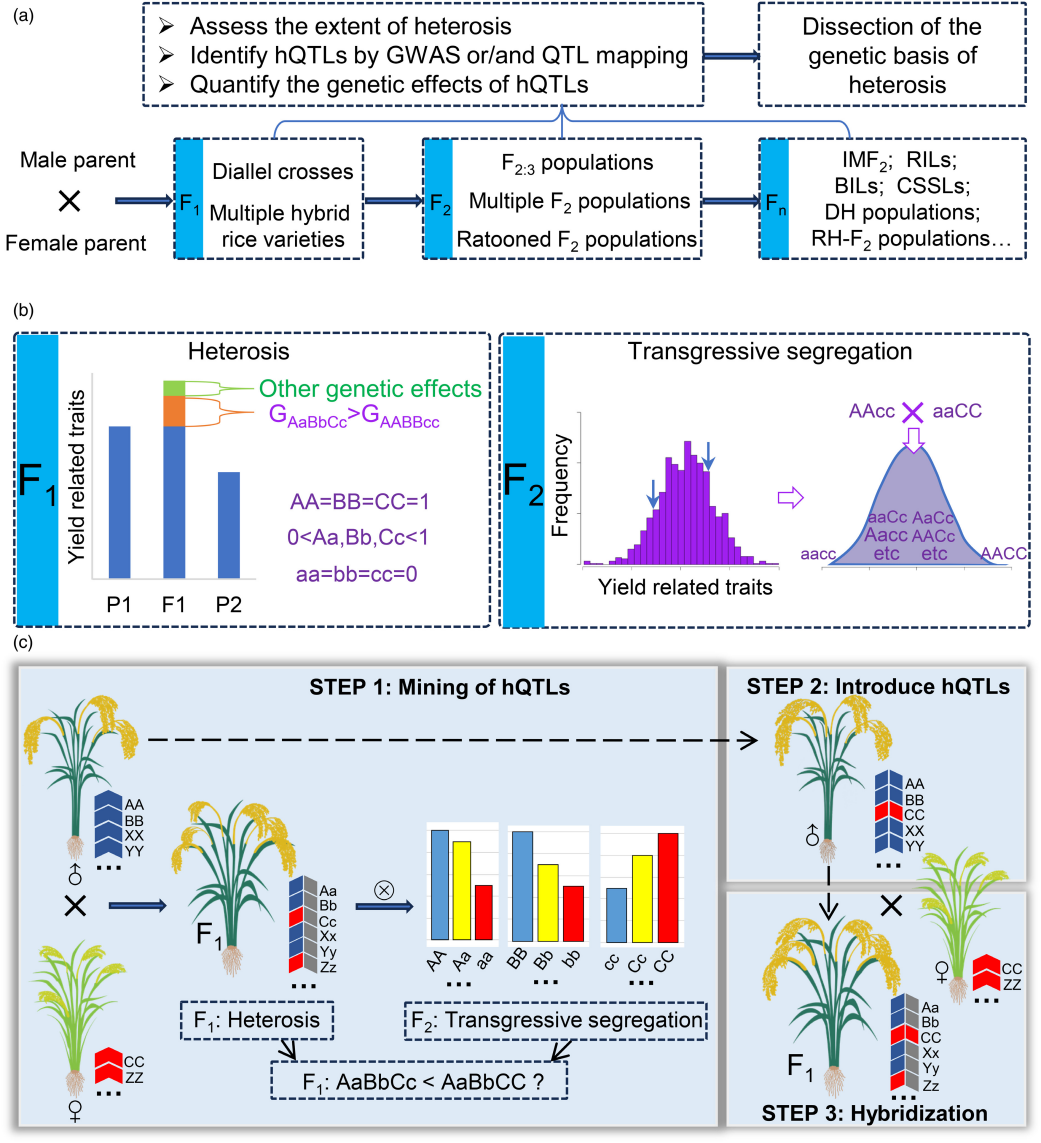
The theoretical basis and roadmap of hybrid breeding strategy. (a) The commonly used genetic populations and approaches for exploring the genetic basis of heterosis. F_1_, first filial generation; F_2_, second filial generation; F_2:3_, F_3_ families derived from a single F_2_ plant; IMF_2_, immortalized F_2_ population; RILs, recombinant inbred lines; BILs, backcross inbred lines; CSSLs, chromosome segment substitution lines; DH, doubled haploid. RH‐F_2_, residual heterozygous − second filial generation. (b) Two schematic diagrams explaining heterosis and transgressive segregation. Left dashed box: The schematic diagram describes the possible contributions of the yield advantage over their corresponding paternal parents. The green part in the bar chart represents yield advantage formed by other genetic effects, such as epistasis and overdominance. The orange part indicates yield advantage formed by dominance effect. The letters A, a, B, b, C, and c indicate favourable alleles at three loci. Upper case letters increase the value of the trait, lower case letters decrease it. The letters A, a, B, and b indicate the alleles at two loci in paternal parent, and C and c represent the allele at one locus in maternal parent. G_AaBbCc_ and G_AABBcc_ indicate the genetic values of the genotypes of ‘AaBbCc’ and ‘AABBcc’ respectively. P1, P2, and F1 indicate the male, female parents and their first filial generation separately. Right dashed box: The histogram on the left represents the common phenomenon of transgressive segregation. The two blue arrows represent the range of parental yield production. Right schematic illustrating a simple model for transgressive segregation. If the beneficial alleles are dispersed among the parents in a cross, it is possible to observe offspring with trait values that are higher or lower than those of the parents. (c) The roadmap for improving breeding strategies through hybridization. ‘F_1_: AaBbCc<AaBbCC?’ indicate the hypothesis being tested in this study, which is whether F_1_ plants with genotype ‘AaBbCC’ (maternal homozygous genotype at one favourable allele) have higher yields compared to those with genotype ‘AaBbCc’ (maternal heterozygous genotype). The letters XX, YY and ZZ represent three additional favourable genes derived from the male and female parents. Similarly, upper case letters increase the value of the trait, lower case letters decrease it.

Relying solely on random hybridization for hybrid breeding is a labour‐intensive and time‐consuming task. A comprehensive understanding of the genetic mechanisms of heterosis could efficiently guide hybrid breeding. To this end, as shown in Figure 1a, various genetic populations were designed to uncover the genetic underpinnings underlying heterosis through the identification of hQTLs and estimation of their effects (Liu *et al*., 2020b; Ouyang *et al*., 2022). Based on different heterotic populations, we have summarized the research on the genetic mechanisms of heterosis in plants over the past few decades. As shown in Table S1, dominance, overdominance, and epistasis contribute to the heterosis, with the dominance effect remaining the primary genetic mechanism.

In our previous work, we employed an integrated quantitative genetics approach to study a large number of hybrid rice varieties and their F_2_ populations, including many time‐honoured elite hybrids with strong heterosis. Our research also revealed that yield‐related heterosis in hybrid rice was primarily attributed to the accumulation of favourable alleles with incomplete dominance, and rare favourable alleles from the female parents contributed significantly to the superiority of the hybrid over their male parents (Gu *et al*., 2023; Huang *et al*., 2015, 2016). Furthermore, heterosis and transgressive segregation are the reasons why plant breeding works (Mackay *et al*., 2021). Although the genetic basis of transgressive segregation appears to be largely distinct from that underlying heterosis, numerous quantitative genetic studies consistently support that the primary cause of both phenomena is the action of complementary genes (deVicente and Tanksley, 1993; Rieseberg *et al*., 1999). We have drawn a simple diagram to explain the genetic basis and underlying connection of heterosis and transgressive segregation. Based on the information depicted in Figure 1b, we consider ‘AA’ and ‘BB’ as favourable alleles from the paternal parent, while ‘CC’ represents superior alleles from the maternal parent, and their heterozygous genotypes exhibit incomplete dominance. Compared to other genetic effects such as overdominance and epistasis, a larger portion of the advantage of hybrids over their male parents is likely caused by dominant effects. Furthermore, the contribution of heterozygous favourable alleles from the maternal parent to the advantage over the male parents, minus the contribution reduced by the paternal favourable alleles' heterozygosity, remains significantly greater than the phenotypic value of the paternal genotype. In other words, for the manifestation of a superior advantage over the paternal parent, the genetic value of ‘AaBbCc’ should exceed that of ‘AABBcc’ (Figure 1b). Certainly, in the F_2_ segregating progeny, there are still many individuals that surpass their parents in certain traits (directional transgressive segregation) and exhibit a phenomenon similar to hybrid vigour observed in F_1_ hybrids (Figure 1b). This ubiquitous phenomenon can also be explained by the dispersion of favourable genes (Mackay *et al*., 2021; Wang *et al*., 2015).

Given all the information above, can we manipulate the maternal heterotic genes to maximize their beneficial properties for yield‐related heterosis in a more directed manner, based on our understanding of their genetic effects? For this purpose, we propose a hybrid breeding improvement strategy. This strategy consists of three main steps (Figure 1c). The first step is to identify the heterotic genes from the female parents. In the F_2_ segregating population of hybrid varieties exhibiting heterotic performance, the incomplete dominant loci in the maternal parent are identified through QTL mapping. The second step involves genetic manipulation through hybrid backcrossing or gene editing to introduce the favourable allele genotypes from the maternal parent into the paternal parent. This manipulation aims to incorporate the advantageous heterotic genes from the maternal parent into the genetic background of the paternal parent. The third step is to cross the improved paternal parent, which now contains the favourable heterotic genes from the maternal parent, with the original maternal parent to obtain improved hybrid rice varieties. This cross will combine the desirable traits of both parents, potentially leading to increased yield or other favourable traits without sacrificing heterotic performance. The subsequent results will validate how this strategy can further enhance yield without compromising heterotic performance.

### Alternative splicing induced by CRISPR‐Cas9 in a heterosis gene *OsMADS1* ^*GW3p6*^ improves the grain yield and quality in rice

We reviewed the mapping results of the F_2_ hybrid rice population of GLY676, which includes both parents, and found that the majority of the QTLs exhibited incomplete dominance (Table S2). Among these loci, the heterotic gene *GW3p6* from the maternal parent not only showed incomplete dominance but also made a significant contribution to yield‐related traits (LOD = 46.25, phenotypic variance = 14.98%). Therefore, *GW3p6* appears to be a promising candidate gene for validating this breeding strategy.

**Figure 2:**
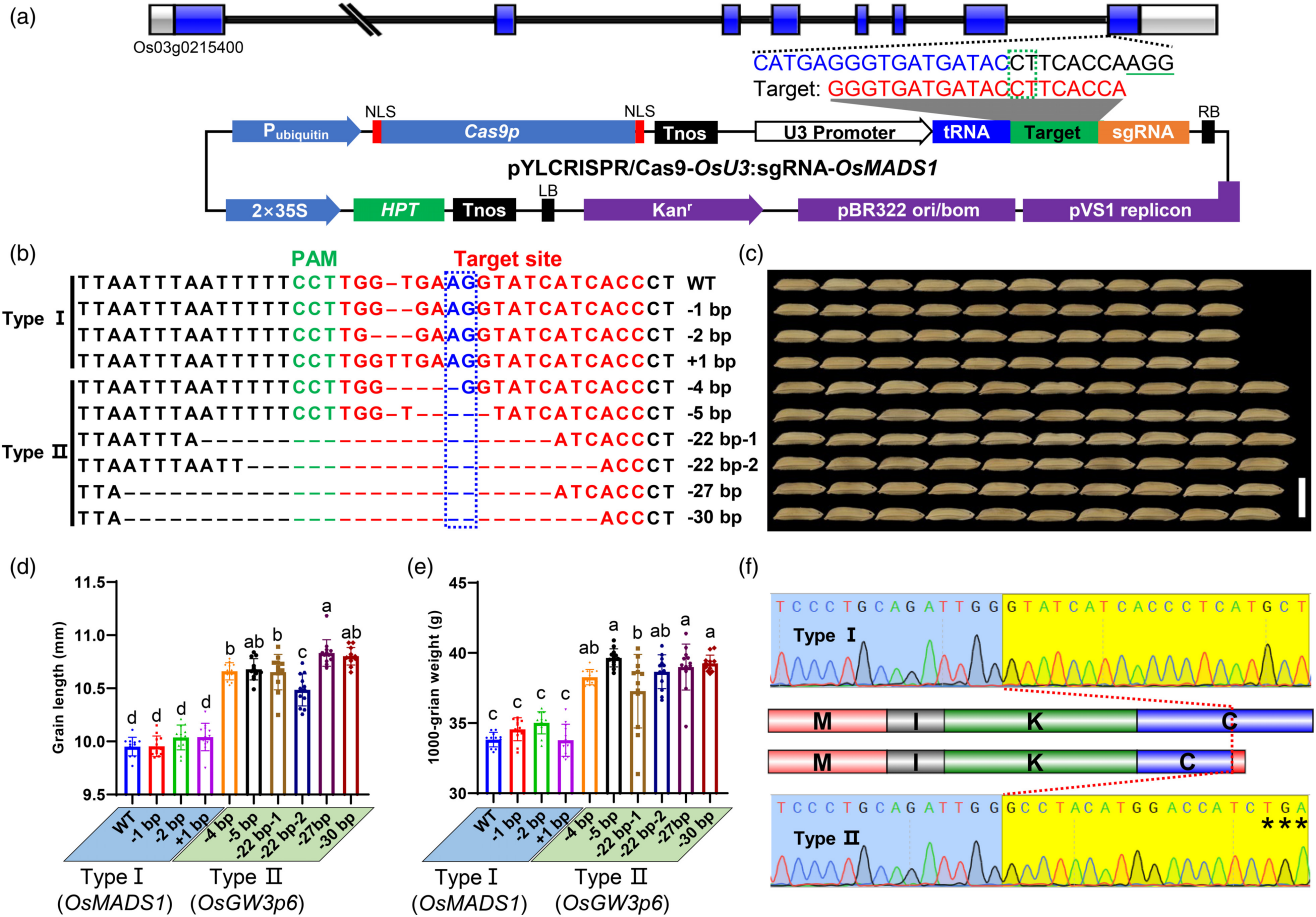
Alternative splicing induced by CRISPR‐Cas9 in *OsMADS1* ^*GW3p6*^ and the grain phenotypes of transgenic plants. (a) Structure of *OsMADS1* and CRISPR‐Cas9 vector. The green dotted box and green line indicate the positions of 3′‐splice site and PAM site, respectively. *HPT* stands for the *hygromycin B phosphotransferase* gene in the CRISPR‐Cas9 vector. (b) Genomic sequence of T_2_ lines of FH676 at the target site. The Arabic numerals and their signs represented the number of inserted or deleted nucleotides. (c) Grain phenotypes of T_2_ lines. Scale bar, 1 cm. (d–e) Statistical analysis of grain length (d) and TGW (e) of T_2_ lines. The letters in parentheses represented the respective mRNAs of *OsMADS1* and *OsMADS1* ^*GW3p6*^. Data shown as mean ± s.d. (*n* = 12 plants). Statistical analyses were performed by Duncan's multiple range tests. The presence of the same lowercase letter denotes a non‐significant difference between means (*P* > 0.05). (f) cDNA sequencing of Type I & II plants. The red dotted line indicates the junction between exon 7 and 8 of *OsMADS1*. The asterisks show the position of the stop codon. The schematic illustration in the middle indicates the functional domains of OsMADS1 (upper) and OsMADS1^GW3p6^ (lower).

*qLGY3*/*OsLG3b*/*GW3p6*, encoding a MIKC‐type MADS‐box transcription factor OsMADS1, strikingly increases the grain length and thus the grain yield in hybrid rice or inbred rice cultivars (Liu *et al*., 2018; Wang *et al*., 2019; Yu *et al*., 2018). A 15‐bp non‐homologous genomic fragment spanning the intron‐exon junction of *OsMADS1* resulted in an alternatively spliced protein, which is associated with the increase in grain length and weight. Moreover, a vast repertoire of mRNA isoforms could be produced through AS of multiexon genes' precursor mRNAs (pre‐mRNAs). Most pre‐mRNAs with GT‐AG splice sites are processed by spliceosomes to generate mature mRNAs by removing introns and joining of exons based on canonical GU‐AG rule (Reddy *et al*., 2013). According to the GU‐AG rule and the genomic position of 15‐bp non‐homologous fragment, we designed a sgRNA targeting the splice acceptor site (dinucleotides AG) at the seventh intron of *OsMADS1* (Figure 2a). The vector *pYLCRISPR/Cas9‐OsU3:sgRNA‐OsMADS1* was introduced into Fuhui676 (FH676, male parent of two‐line elite hybrid rice GLY676) through *Agrobacterium*‐mediated transformation.

By sequencing the PCR amplicons of the non‐homologous genomic fragment and selecting hygromycin B (hyg)‐resistant plants, we obtained six T_0_ transgenic plants comprising uniform bi‐allelic and heterozygous mutations. Ultimately, over 40 individual T_2_ transgenic lines were examined to screen for homozygous mutations and investigate the phenotypic effect of the mutants. A total of 10 genotypes exist in all T_2_ lines, including one with a 1 bp insertion and the others with various deletions (Figure 2b). Based on whether the 3′ acceptor site (AG) was disrupted or not, we divided these genotypes into two types: Type I and Type II. Type I plants, harbouring the original splice site, exhibited similar grain length and thousand‐grain weight (TGW) to the transgenic negative lines (WT). But compared to Type I plants, the grain length and TGW of Type II plants were increased by 6.9% and 12.8%, respectively (Figure 2c–e). Unlike Type I with original mature mRNA of *OsMADS1*, 3′RACE and cDNA sequencing revealed that Type II plants produced an alternative 3′ splicing event: the splice site shifted to the 32nd nucleotide (AG/GC) of exon 8 of *OsMADS1*, causing a premature termination codon and a truncated exon 8 (Figure 2f and Figure S1). This AS isoform is identical to that of *qLGY3*/*OsLG3b*/*GW3p6*, indicating that the alternatively spliced protein of OsMADS1 is truncated by 32 amino acid residues to influence its C‐terminal domain (Liu *et al*., 2018). All these results suggested that AS of *OsMADS1* induced by CRISPR‐Cas9 system can significantly increase grain length and grain weight by disrupting the 3′ splice site.

### The Type II genotype could simultaneously improve grain yield and quality with no apparent trade‐off in other agronomic traits

**Figure 3:**
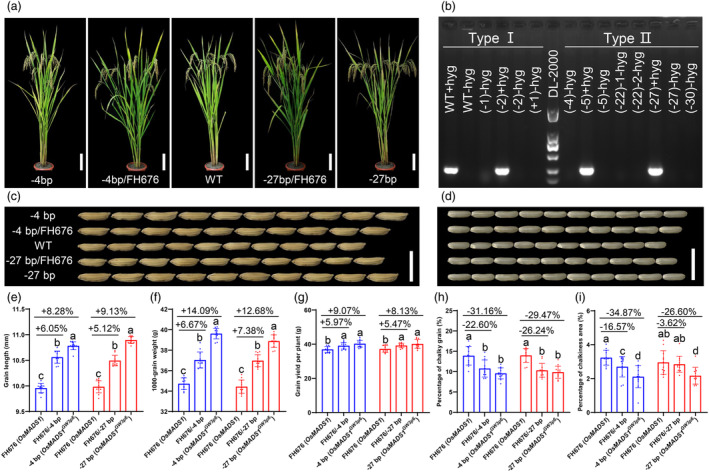
The phenotype of grain size and quality in FH676 transgene‐free plants. (a) Comparison of plant morphology in BC_2_F_2_ plants. Scale bars = 20 cm. (b) PCR amplicons of hyg resistance gene in BC_2_F_2_ and T_2_ plants. All positive plants with the hyg‐B‐resistance gene are conducted in T_2_ lines. (c–i) Grain morphology (c), brown rice (d), grain length (e), TGW (f), grain yield per plant (g), percentage of chalky grain (h), and chalkiness area (i) in FH676 and BC_2_F_2_ plants with the heterozygous and homozygous genotypes of Type II. The letters in parentheses represent the same mRNA as *OsMADS1* and *OsMADS1* ^*GW3p6*^. Data shown as mean ± s.d. (*n* = 12 plants). Statistical analyses were performed by Duncan's multiple range tests. The presence of the same lowercase letter denotes a non‐significant difference between means (*P* > 0.05).

As the genome editing vectors can be segregated out from mutant genomes through crossing or selfing, we crossed T_2_ plants with FH676 to screen for transgene‐free mutant plants, and thus obtained transgene‐free plants in the BC_2_F_2_ progeny (Figure 3a). Neither the genomic fragment of hpt (hygromycin B phototransferase) nor cas9 protein was detected in these transgene‐free plants (Figure 3b and Figure S2). Phenotypic comparison of the two types of plants indicated that Type II plants had remarkable increases in grain length (8.5%), TGW (13%), and grain yield per plant (9.2%) compared to FH676 and Type I plants (Figure 3c–g and Figure S3), whereas other important agronomically traits did not change significantly (Figure 3a and Figure S4). *OsMADS1* ^*GW3p6*^, a heterosis gene derived from the female parent, explained a large proportion in the yield related better‐parent heterosis in the two‐line hybrid rice GLY676, and its heterozygous state displayed a positive partial dominance effect (Huang *et al*., 2016; Wang *et al*., 2019). We therefore crossed BC_2_F_2_ plants with FH676 to evaluate the genetic effects of heterozygous *OsMADS1*. As shown in Figure 3c–g, the genetic effects of heterozygous Type II alleles showed a partial positive dominance effect, which is consistent with previous findings (Huang *et al*., 2016; Wang *et al*., 2019). This result further supported the notion that the heterozygous state of the heterotic gene *OsMADS1* ^*GW3p6*^ could greatly enhance grain yield. *OsMADS1* ^*lgy3*^ is responsible for better appearance quality in terms of grain length‐to‐width ratio and grain chalkiness (Liu *et al*., 2018), and recent study has shown that *OsMADS1* affects grain quality by regulating gene expressions and regulatory networks of starch and storage protein metabolisms in rice grains (Liu *et al*., 2023). Therefore, we used image analysis systems and scanners to analyse the grain appearance quality of the gene‐edited BC_2_F_2_ plants. We observed a slight difference between Type I and Type II plants in grain chalkiness except for grain length‐to‐width ratio (Figure S5). The percentage of endosperms with chalkiness and the degree of endosperm chalkiness of Type II plants are approximately 4.4% (about 30% decrease) and 0.95% (about 31% decrease) lower than that of Type I plants, respectively (Figure 3h,i and Figure S5). Intriguingly, the plants with a heterozygous state of *OsMADS1* ^*GW3p6*^ also exhibit partial positive dominance for the phenotype of chalky grain and chalkiness area (Figure 3h,i). Further comparisons of endosperm transparency showed no significance difference between the two types of *OsMADS1* (Figure S5). Collectively, the CRISPR‐Cas9‐mediated AS isoform of *OsMADS1* played dual roles in increasing grain yield and quality. More importantly, these transgene‐free restore lines with type II genotypes, coupled with this gene‐editing target site that effectively caused AS of *OsMADS1*, hold tremendous promise in rice breeding, especially in improving the male parent in hybrid rice breeding.

### Introducing Type II genotype into hybrid rice GLY676 could further boost grain yield

Extensive genomic analyses of hybrid rice varieties have revealed that the heterozygosity of whole‐genome genotypes has a limited contribution to yield‐related heterosis through in‐depth analyses of the effects of heterozygous genotypes, but a strong correlation is observed between the yield and the accumulation of superior alleles (Huang *et al*., 2015; Yang *et al*., 2017a, 2017b). Since the genetic effect of *OsMADS1*/*OsMADS1* ^*GW3p6*^ is partial positive dominance in grain yield (Figure 3c–g), it is very promising to manipulate heterosis genes to break the yield ceiling of current hybrid rice varieties.

**Figure 4:**
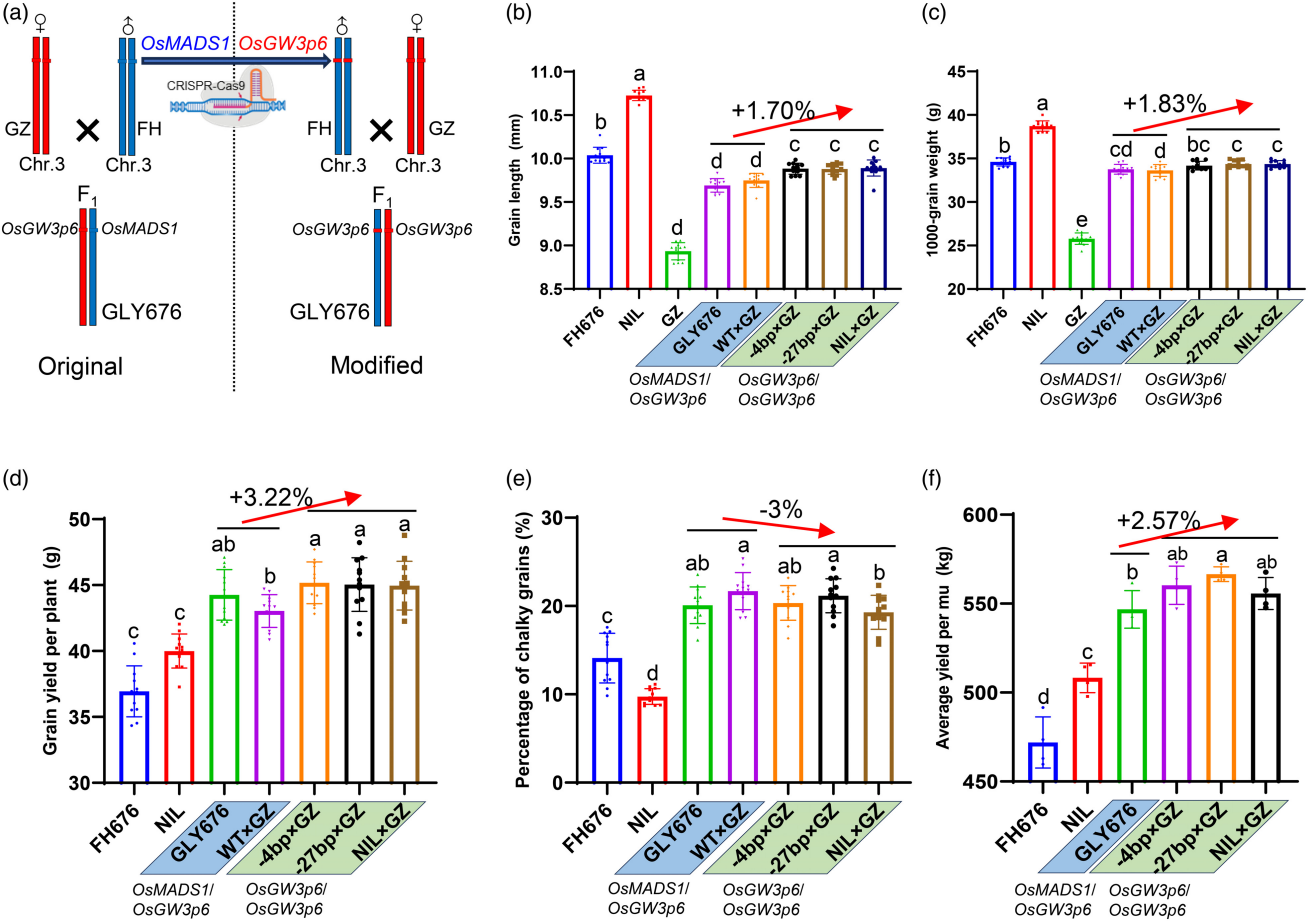
The performance of grain yield and grain quality among various hybrid combinations of GLY676. (a) The schematic diagram shows the genotype of chromosome 3 of the new hybrids. FH and GZ indicate the male and female parents of GLY676, respectively. The colours of blue and red represent the genotypes of the male parent and female parent, respectively. The heterozygous genotype of the F_1_ progeny (GLY676) of FH and GZ is represented by red and blue boxes arranged side by side. The arrowhead indicates using CRISPR‐Cas9 to change *OsMADS1* to *OsMADS1* ^*GW3p6*^ in FH676 plants. (b–f) Statistical analyses of grain length (b), TGW (c), grain yield per plant (d), percentage of chalky grain (e), and average yield per mu (f) among different hybrid combinations of GLY676. The genotypes below represent the same haplotypes present in these genetic materials. Values indicate means ± s.d. Exact *P* values are performed by Duncan's multiple range tests. (b–e) *n* = 12 plants. (f) *n* = 4 paddies; each paddy contained 480 plants in the 20.6‐m^2^ field. mu, 666.66 m^2^ or a fifteenth hectare.

Therefore, we crossed Type II plants of FH676 with the male sterile line Guangzhan63‐4S (GZ) to produce new hybrids (Figure 4a). Multiple molecular markers distributed on each chromosome are used to check the heterozygosity of new rice hybrids (Figure S6a). Genotyping analysis indicated the purity of new hybrid seeds is 100%, which ensures the same genetic background as the GLY676 (Figure S6b–d). Although the grain length and TGW of the new F_1_ hybrids exhibited mid‐parent heterosis (less than FH676), their grain length and TGW are still higher than GLY676 and the plants of WT (Type I) × GZ (Figure 4b,c). Compared to FH676, the plants of WT (Type I) × GZ, GLY676, and the new hybrids (homologous genotype of *OsMADS1* ^*GW3p6*^) exhibited higher grain yield per plant, accounting for an average of 16.5%, 19.8%, and 22.1%, respectively (Figure 4d). We found no obvious differences in grain appearance quality among these hybrid combinations (Figure 4e), indicating that increasing the dosage of *OsMADS1* ^*GW3p6*^ (from heterozygous to homozygous state) could improve the yield of hybrid rice without an apparent trade‐off in grain quality. Intriguingly, over three successive years of field trialling, GLY676 and the new hybrids out‐yielded FH676 by an average of 11.24% and 13.16%, respectively (Figure 4f, Figure S7). Similarity, a new hybrid generated by crossing a near‐isogenic line of *OsMADS1* ^*GW3p6*^ in FH676 genetic background (NIL) with GZ had comparable grain yield performance with other new modified hybrids carrying the homologous *OsMADS1* ^*GW3p6*^ genotype (Figure 4d,f). Thus, we produced the improved hybrids with practical potential in rice yield. These results also prove that our hybrid breeding strategy is effective and lend credence to the view that dominance effect underlying functional complementation is an indispensable contributor to yield heterosis.

### The increased yield in three‐line hybrid rice by assimilating a heterosis gene demonstrated an effective and time‐saving strategy for hybrid breeding

**Figure 5:**
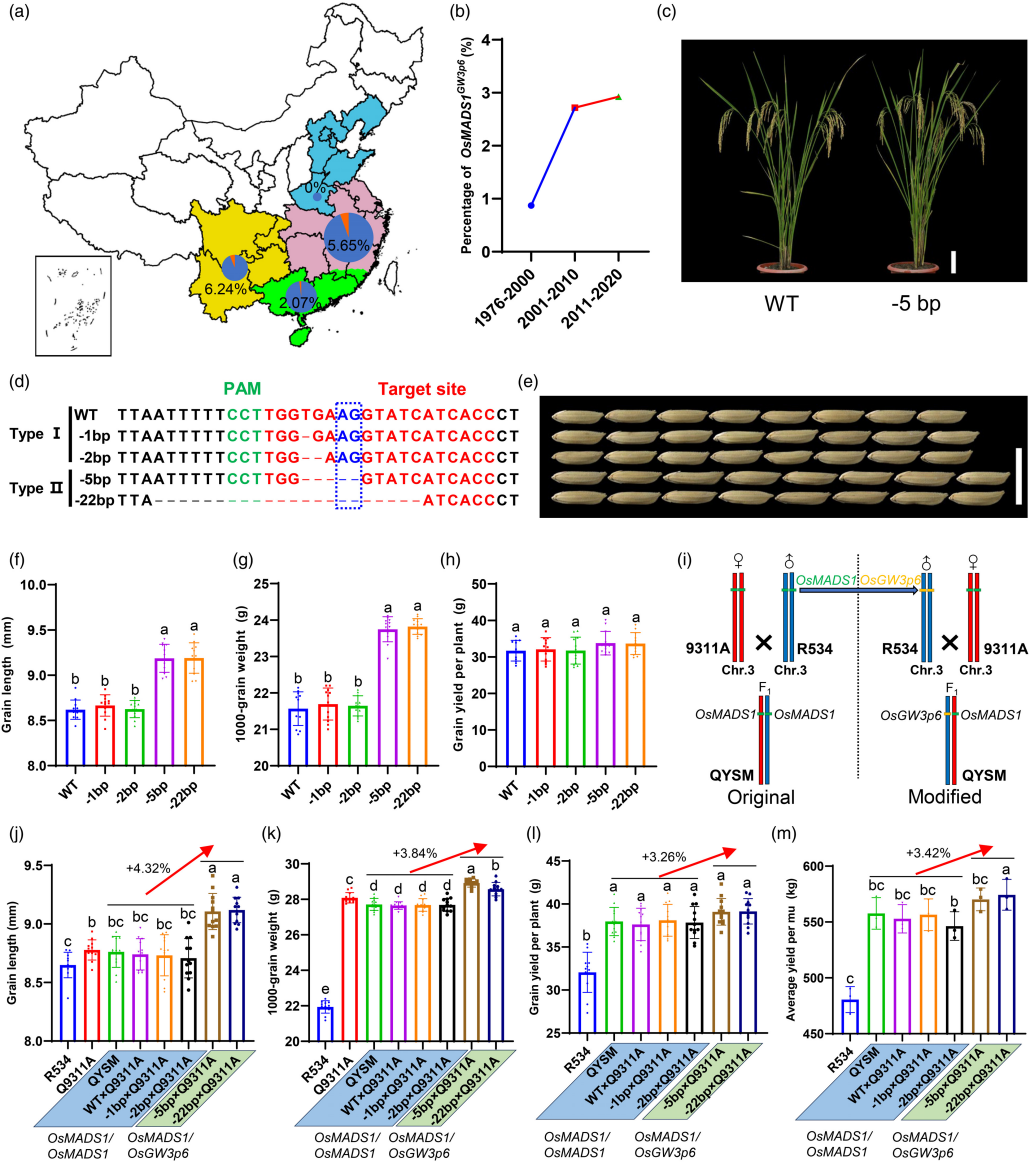
Introducing a heterosis gene into hybrid rice QYSM showed great potential for yield increase. (a) The geographic distribution of the hybrid rice accessions containing *OsMADS1* and *OsMADS1* ^*GW3p6*^ alleles. The colour of blue and orange in the pie graph represent the haplotypes of *OsMADS1* and *OsMADS1* ^*GW3p6*^, respectively. The values closed to the pie graph represent the proportion of *OsMADS1* ^*GW3p6*^ haplotype in this area. The size of the pie graph indicates the number of hybrid rice varieties. (b) The proportion of the haplotype of *OsMADS1* ^*GW3p6*^ in different breeding periods. (c) Gross morphologies of transgenic plants with the genotypes of Type I and Type II. (d) The genotypes of T_2_ plants of R534 at the target site of *OsMADS1*. (e–h) Grain size and shape (e), grain length (f), TGW (g), and grain yield per plant (h) in transgenic T_2_ plants of R534. *n* = 12 plants. (i) The illustration represents the genotypes of the hybrid rice QYSM and its parents in chromosome 3 and exhibits the general process of the strategy for hybrid breeding by introducing a heterosis gene. Blue and red indicated the genotypes of male parents and female parents, respectively. The green and orange boxes indicate the genotypes of *OsMADS1* and Type II (or *OsMADS1* ^*GW3p6*^), respectively. The heterozygous genotypes of *OsMADS1* (*OsMADS1*/Type II or *OsMADS1*/*OsMADS1* ^*GW3p6*^) in modified QYSM are represented by green and orange boxes arranged side by side. (j–m) Statistical analyses of grain length (j), TGW (k), grain yield per plant (l), and average yield per mu (m) in various hybrid combinations of QYSM. The genotypes below represent the same haplotypes present in these genetic materials. (j–k) Data shown as mean ± s.d. (*n* = 12 plants). (m) *n* = 3 paddies, each paddy contained 240 plants in the 10.66‐m^2^ field. mu, 666.66 m^2^ or a fifteenth hectare. Statistical analyses were performed by Duncan's multiple range tests.

To test if this approach that introduces a heterosis gene *OsMADS1* ^*GW3p6*^ into the parental plants to enhance grain yield has great potential in hybrid rice breeding, we first perform haplotype analysis to find the proportion of the haplotype of *OsMADS1* ^*GW3p6*^ in 2932 commercial hybrid rice varieties. The results showed that approximately 5.25% of the 2932 hybrid rice varieties carried the haplotype of *OsMADS1* ^*GW3p6*^ (Dataset S1). Subsequently, we collected 566 hybrid rice parents, consisting of 407 male parents and 159 female parents, to investigate the frequency of *OsMADS1* ^*GW3p6*^ application in all parents of hybrid rice (Dataset S2). About 3.36% of the parent materials possessed the haplotype of *OsMADS1* ^*GW3p6*^, and the ratio in female parents (8.18%) far outstrips that in male parents (1.47%). Intriguingly, only three varieties with the homozygous genotype of *OsMADS1* ^*GW3p6*^ occur in 2932 hybrid rice varieties, which may be associated with a lower possibility for the homozygous genotype of *OsMADS1* ^*GW3p6*^ in the progeny formed by crossing their parents harbouring the haplotype of *OsMADS1* ^*GW3p6*^. In addition, diverse hybrid rice varieties containing geographic and chronological information enable us to examine the dynamic utilization process of *OsMADS1* ^*GW3p6*^. We found that the haplotype of *OsMADS1* ^*GW3p6*^ mainly existed in three planting areas: the middle and lower reaches of the Yangtze River, southwestern China, and Southern China (Figure 5a). Among these areas, the highest proportion of the haplotype in southwestern China may be relevant to the breeding concept − heavy panicle and large grain size of hybrid rice − pursued by breeders in this region. According to the release time of these hybrid rice, we divided them into three periods: 1976–2000, 2001–2010, and 2011–2020. Haplotype analysis showed a noticeable increase with time in the proportion of *OsMADS1* ^*GW3p6*^ (Figure 5b). All these data indicated that *OsMADS1* ^*GW3p6*^ is a rare superior allele that has not yet been widely used and has excellent potential in hybrid rice breeding.

To further validate the feasibility of this approach, we tried to modify *OsMADS1* in other representative hybrid rice through the CRISPR‐Cas9 system. Wu‐shan‐si‐miao (R534) is an inbred rice variety featuring high yield and grain quality and is widely used as a restorer line for two‐line and three‐line hybrid rice due to its high combining ability. Therefore, R534 carrying the haplotype of *OsMADS1* was selected as the candidate plant for genome editing. In proof‐of‐concept experiments, we obtained a total of 17 T_0_ transgenic lines, and four genotypes were generated in all transgenic lines of R534 through Sanger sequencing (Figure 5c,d). Comparison of plant phenotype showed that the genome‐edited plants with Type II genotype increased the grain length, TGW, and grain yield per plant by 6.40%, 9.94%, and 5.89%, respectively, without an apparent negative impact on other agronomic traits (Figure 5e–h, Figure S8). These data also indicated that the genotype of Type II created by CRISPR‐Cas9 technology had great potential for yield improvement in R534.

Quan‐you‐si‐miao (QYSM), a three‐line high‐yield hybrid rice variety, is bred by crossing R534 and the male‐sterile line Quan‐9311A (Q9311A). The genetic background of QYSM is heterozygous, but we found it had a homozygous genotype of *OsMADS1* through Sanger sequencing. For the purpose of crop improvement, we made multiple hybrids to test their yield performance (Figure 5i). In terms of grain length and TGW, we found QYSM and new hybrid combinations with homozygous haplotype of *OsMADS1* (WT × Q9311A, −1 bp × Q9311A, and −2 bp × Q9311A) exhibited mid‐parent heterosis, but other new combinations with heterozygous haplotype of *OsMADS1* ^*GW3p6*^ (−5 bp × Q9311A, and −22 bp × Q9311A) showed better‐parent heterosis (Figure 5j,k). Compared with the hybrids with the homozygous haplotype of *OsMADS1*, the F_1_ hybrids harbouring the heterozygous haplotype of *OsMADS1* ^*GW3p6*^ increased the grain length and TGW by 4.32% and 3.84%, respectively (Figure 5j,k). Besides, the new hybrids with Type II genotype produced higher grain weight than QYSM and Type I hybrids, thus resulting in a 3.26% increase in grain yield per plant compared with the QYSM and the hybrids with Type I genotype (Figure 5l). More importantly, the field test indicated that the plants with Type II genotype, on average, had an 3.42% increase compared with QYSM and Type I hybrids (Figure 5m). All these data suggested that this approach could quickly improve grain yield in QYSM, implying great potential in hybrid rice breeding.

## Discussion

Despite the emergence about various genetic models to explain the genetic basis of heterosis, little consensus has yet been reached. More studies provided empirical support for the dominance hypothesis compared to other genetic models (Li *et al*., 2016; Shen *et al*., 2022; Yang *et al*., 2017a). For example, the hybrids pyramiding multiple heterotic loci in the female or combined parental genome background showed comparable yield performance to that of the hybrid rice variety Shanyou63 (Shen *et al*., 2022). This result demonstrated that the dominance effect played an important role in yield heterosis, and yield‐related heterosis could be achieved by manipulating several major dominant heterotic loci. Previous research has revealed that *OsMADS1* ^*GW3p6*^ is a typical heterosis locus that can significantly increase grain yield (Wang *et al*., 2019). Moreover, the genetic effect of heterozygous *OsMADS1*/*OsMADS* ^*GW3p6*^ exhibited incomplete dominance (Figure 3c–g), and introducing the heterosis gene *OsMADS1* ^*GW3p6*^ into restorer lines greatly enhanced the grain yield without an apparent negative impact on other agronomic traits (Figure 3g, Figure S4). The above characters of *OsMADS1* ^*GW3p6*^ ensure a higher yield by pyramiding a dominant heterotic gene in hybrid rice. Additionally, this study provided solid evidence that the grain yield of the hybrids with the homozygous genotype of *OsMADS1* ^*GW3p6*^ (or Type II) was higher than that of the hybrids with the heterozygous genotype of *OsMADS1* ^*GW3p6*^ in hybrid rice GLY676, implying dominance model played a leading role in yield‐related heterosis. In the current scenario of similar and saturated combinations of complementary genotypes in hybrid rice, these results demonstrate the feasibility of the breeding strategies that aim to maximize the contribution of heterosis genes in the female parent to further enhance yield. Because it is challenging to obtain genetically modified seeds of Q9311A owing to extremely low fertility, we did not achieve homozygous genotype of *OsMADS1* ^*GW3p6*^ (or Type II) in QYSM. Nevertheless, the results of increased yield in the modified F_1_ of QYSM carrying heterozygous *OsMADS1* ^*GW3p6*^, demonstrated that accumulating heterozygous heterosis gene with incomplete dominance can still improve hybrid rice. It is two sides of the same coin of our hybrid breeding strategy. In the future, we will introduce the Type II genotype into the maintainer line Q9311B, which will help us obtain a new QYSM with a homozygous Type II genotype to further investigate the extent of increase in grain yield.

Our study also showed the potential of single heterosis gene to increase rice production in hybrid rice. For the trait of grain yield per plant, *OsMADS1* ^*GW3p6*^ (or Type II genotype) in hybrid combinations of GLY676 and QYSM explained over 40% and over 30% of the yield advantage of hybrids over their male parents, respectively (Data S1). This data proved the genomic loci from the female parents contributed a lot to better‐parent heterosis of rice yield. As genomic insights into rice heterosis have suggested that the heterozygous state of heterotic loci mostly acted through the way of positive partial dominance, we observed a remarkable growth in grain yield by introducing Type II genotype into QYSM even though the genotype of *OsMADS1* ^*GW3p6*^ in QYSM is heterozygous. Meanwhile, we found that the proportion of the haplotype of *OsMADS1* ^*GW3p6*^ is low in 2932 hybrid rice varieties, which is because *OsMADS1* ^*GW3p6*^ may have no effect in other genetic backgrounds. These results also inspire us that hQTLs from male parents, including *OsMADS1* ^*GW3p6*^, should be introduced into more hybrid rice varieties to verify whether this approach can further improve grain yield. In this study, we have achieved an increase in grain yield in two representative two‐line and three‐line hybrid rice varieties (Figure 4d,f; Figure 5l,m). Numerous published studies on the genome analysis of hybrid rice have shown that the breeders introduced different exogenous genomes of other rice subpopulations to construct male and female parents of hybrid rice, thereby shaped the genome landscape of heterosis in hybrid rice (Gu *et al*., 2023; Lin *et al*., 2020). Therefore, we inferred from these results that the approach of introducing *OsMADS1* ^*GW3p6*^ into hybrid rice would be effective in most hybrid rice varieties.

Yield‐related heterosis is characterized by a substantial increase in yield compared to the parental lines, which is attributed to the rearrangement of genotypes (or hQTLs) inherited from the parents. Among these hQTLs, many heterosis genes exhibit pleiotropy in important agronomic traits, such as the major hQTL *DTH8* with pleiotropic effects on grain yield, heading date, and plant height (Yan *et al*., 2011). And previous studies also shown that several major heading date genes determined strong heterosis of commercial rice hybrids in diverse ecological regions (Huang *et al*., 2016; Zhou *et al*., 2021). In this study, both FH676 and R534 carrying the Type II genotype showed a slight increase in heading date compared to the wild type (Figures S4b and S8b). However, we did not observe significant differences in heading date among the improved F_1_ hybrids (Figure S9). Considering the potential inconvenience in agricultural production and the adaptation to ecological regions caused by significant changes in heading date, *OsMADS1* ^*GW3p6*^, which exhibits less pronounced phenotypic variations in heading date, remains highly valuable in breeding.

Genome editing is a powerful and prevalent method in crop improvement and is set to revolutionize plant breeding. Our study reflected several advantages in harnessing CRISPR‐Cas9 to edit a heterosis gene for cross breeding. First, our approach is time‐ and labour‐saving. Compared to the tedious work of conventional cross breeding, we estimated that this approach could save at least 50% of time and labour costs for hybrid rice breeding. The extensive use of DNA‐free genome editing methods to obtain transgene‐free mutant parents will further accelerate the process of hybrid rice breeding (Liang *et al*., 2017; Svitashev *et al*., 2016; Zhang *et al*., 2016). Second, introducing hQTLs into hybrid rice could effectively prevent the genetic drag between heterosis genes and deleterious genes. Precise gene editing enables the optimization of agronomic traits in hybrid rice without damaging their unique genetic backgrounds. In addition, previous studies have revealed that *OsMADS1* ^*GW3p6*^ originated from tropical japonica (Liu *et al*., 2018; Wang *et al*., 2019; Yu *et al*., 2018). So, it may be difficult to effectively utilize this gene due to linkage drag, resulting in a low proportion of *OsMADS1* ^*GW3p6*^ in 2932 hybrid rice varieties. This study showed massive potential for crop improvement by fine‐tuning the genome of hybrid rice. Third, our breeding strategy with good versatility will be applicable to combine desirable traits in hybrid rice breeding. For example, a three‐line hybrid variety with increased grain aroma could be produced by introducing new alleles of *BADH2*, demonstrating a similar strategy to our approach (Hui *et al*., 2022). In view of this, some essential QTLs that control certain agronomic traits without a trade‐off effect in other traits will be suitable targets for precisely modifying traits in hybrid rice. Additionally, the ever‐increasing known quantitative trait nucleotides integrating with various genome editing tools will further expand the scope of rapid genetic improvement in hybrid breeding (Wei *et al*., 2021a). Further research should quantify the genetic effect of more hQTLs and still need to verify the increasing effect in agronomic traits by editing the candidate hQTLs in hybrid rice (Mackay *et al*., 2021; Wang and Han, 2022). We believed that the total grain output of the genome‐edited parental inbred lines could exceed that of their hybrid rice by introducing multiple heterosis loci in the future.

In conclusion, we achieved an alternative 3′ splicing event of *OsMADS1* by disrupting the 3′ splice site using the CRISPR‐Cas9 system. We also observed that AS of *OsMADS1* could simultaneously enhance rice yield and grain quality. Also, the new hybrids harbouring homozygous Type II genotypes or *OsMADS1* ^*GW3p6*^ under the same hybrid genetic backgrounds have a better performance in grain yield compared to the hybrid variety GLY676, which demonstrates that our hybrid breeding strategy is a promising and time‐saving strategy for breeding higher‐yielding rice varieties beyond what is currently achievable. Our research also showed great potential of single heterosis gene from the female parents in increasing grain yield of hybrid rice. Continuous improvements in both rice yield and grain quality are long‐term goals to cope with the challenges of population growth and climate change. With the development of rice functional genomics and powerful genome editing technologies, increasing superior alleles could be harnessed in rice breeding (Wang and Han, 2022). Overall, our study provided a valuable breeding target and an efficient method for hybrid rice breeding.

## Materials and methods

### Plant materials and growth conditions

The *indica* varieties FH676, GZ63‐4S, NIL‐*GW3p6*, GLY676, R534, Q9311A, and QYSM were used in this study. All these plants, including transgenic and non‐transgenic plants, were planted in the experimental fields of the CAS Center for Excellence in Molecular Plant Sciences in Shanghai and Hainan, China, from 2018 to 2023. Twelve 30‐day‐old seedlings of each line were transplanted with 17 cm spacing in single row plots in the rice paddy field, and rows were 30 cm apart.

### Phenotypic evaluation

Fully filled grains on each plant were used to measure grain length, grain width, thousand‐grain weight, and yield per plant. And the grain size of rice was measured by an automatic digital grain scanner, and the statistics of grain length and width were conducted from the image analysis software (Wseen SC‐G). Ten plants from the middle of each row were harvested individually for trait measurement. The phenotypic data of grain number per panicle, panicle length, and seed setting rate was obtained from the three longest panicles of each plant.

The percentage of chalky grains was scored by the visual assessment of the percentage of white‐core grains in random samples of more than 100 dehulled grains from each plant. The degree of endosperm chalkiness was obtained from image processing software (Wseen SC‐E) by scanning the milled rice.

To obtain the total yields of field‐grown rice, we planted different types of rice varieties in the same paddy fields in parallel. For all rice varieties of GLY676 and QYSM, the average planting density of rice is about 15 152 plants per mu. 480 and 240 plants of every rice variety grown in the 20.6‐m^2^ and 10.65‐m^2^ paddies were harvested, respectively. Threshed seeds from each plant were air dried and stored at room temperature for 2 months before further weigh. The seed purity of F_1_ hybrids was confirmed by multiple molecular markers.

### Plasmid construction and plant transformation

According to the genomic position of the 15‐bp non‐homologous fragment, we designed a sgRNA targeting the splice acceptor site at the seventh intron of *OsMADS1*. The sgRNA target of CRISPR‐Cas9 was designed on the website: http://crispr.hzau.edu.cn/CRISPR2. A 20‐bp target region was inserted into the intermediate vector pYLgRNA‐OsU3/*LacZ*, and the integrated U3‐target‐gRNA region was cloned and subsequently inserted into the vector pYLCRISPR/Cas9‐MH.

The constructs were introduced into *Agrobacterium tumefaciens* strain EHA105 and transferred into the FH676 and R534 by *Agrobacterium*‐mediated transformation. The primers used in this study are listed in Table S3.

### Screening of transgene‐free plants

The coding sequence of hpt and Cas9 protein were selected as the indicator of exogenous genomic fragments. We designed one and two pairs of primers targeting *hpt* and *cas9*, respectively. After PCR analysis, the plants without *hpt* and *cas9* genome fragments could be identified as transgene‐free plants. In T_2_, BC_1_F_1_, BC_2_F_1_, and BC_2_F_2_ lines of FH676, we screened transgene‐free plants through PCR analysis of three molecular markers. Since T_2_ generation, the BC_2_F_2_ plants of FH676 that have been negative for three genomic fragments in all their generations could be further confirmed as transgene‐free plants.

### RACE and cDNA analysis

The leaves from 2‐week‐old seedlings were harvested and grinded, and the total RNA of rice leaves was extracted with Trizol reagent (Thermo Fisher Scientific, 15 596 026). The genomic DNA removal and first‐strand cDNA synthesis were conducted using ReverTra Ace qPCR RT Master Mix with gDNA Remover (Toyobo, FSQ‐301). The genomic fragments containing the seventh and eighth exons of *OsMADS1* were amplified and sequenced to identify the AS. The full‐length transcripts of *OsMADS1* and *OsMADS1* ^*GW3p6*^ were determined by rapid amplication of cDNA ends (RACE). The cDNA was synthesized following the protocol provided in SMARTer RACE 5′/3′ kit (Takara, 634 858/634859). The primers used for RACE AND cDNA analysis are listed in Table S3.

#### Statistical analysis

Statistical tests were performed by GraphPad Prism 8 and SPSS Statistics 23. All values are reported as mean ± s.d.

## Competing of interests

The authors declare no competing interest.

## Author contributions

C.W. performed most of the experiments, Z.W. and C.W. contributed the generation of the hybrid combinations. C.W., Z.W., Y.C., L.H., Y.W., D.Y., W.Lv., and K.L. characterized the genotypes and phenotypes of the gene‐edited plants. C.W., Z.Z., Q.Z., and L.S. analysed the data. C.W., and B.H. conceived and designed experiments and wrote the manuscript. B.H. directed the research.

## Supporting information


**Dataset S1** The haplotype of OsMADS1 in hybrid rice.

**Dataset S2** The haplotype of OsMADS1 in the parents of hybrid rice.

**Data S1** Phenotypic source data in this study.

**Figure S1** The electropherograms of cDNA sequencing in T2 transgenic plants.
**Figure S2** PCR amplicons of *Cas9* in BC2F2 and T2 plants.
**Figure S3** Statistical analyses of important agronomic traits in BC2F2 lines of FH676 with Type I genotypes.
**Figure S4** Comparisons of agricultural traits in BC2F2 plants.
**Figure S5** Grain appearance in BC2F2 plants.
**Figure S6** Purity identification of hybrid rice seeds.
**Figure S7** The average yield per mu among different hybrid combinations of GLY676 in Hainan and Shanghai.
**Figure S8** Comparisons of agronomic traits in transgenic plants of R534.
**Figure S9** Heading date in multiple hybrid combinations of GLY676 and QYSM.

**Table S1** Progress of the genetic basis of heterosis in plants.

**Table S2** QTL mapping results in GLY676 F2 population.

**Table S3** Primers and probes used in this study.

## Data Availability

All data supporting the findings of this study are available in the article, supplementary information, and Data S1. Source data are provided in this paper.
