# Supplementary material for: A higher‐yield hybrid rice is achieved by assimilating a dominant heterotic gene in inbred parental lines
Source: Plant Biotechnol J. 2024 Mar 7;22(6):1669–80. doi: 10.1111/pbi.14295 (PMC11123404; doi:10.1111/pbi.14295)
Supplement: Supplementary file 4 — Figure S1 The electropherograms of cDNA sequencing in T2 transgenic plants. Figure S2 PCR amplicons of Cas9 in BC2F2 and T2 plants. Figure S3 Statistical analyses of important agronomic traits in BC2F2 lines of FH676 with Type I genotypes. Figure S4 Comparisons of agricultural traits in BC2F2 plants. Figure S5 Grain appearance in BC2F2 plants. Figure S6 Purity identification of hybrid rice seeds. Figure S7 The average yield per mu among different hybrid combinations of GLY676 in Hainan and Shanghai. Figure S8 Comparisons of agronomic traits in transgenic plants of R534. Figure S9 Heading date in multiple hybrid combinations of GLY676 and QYSM. [file PBI-22-1669-s007.pdf]

Supplemental information

Figure S1. The electropherograms of cDNA sequencing in T_2 transgenic plants.

Figure S2. PCR amplicons of *Cas9* in BC_2F_2 and T_2 plants.

Figure S3. Statistical analyses of important agronomic traits in BC_2F_2 lines of FH676 with Type I genotypes.

Figure S4. Comparisons of agricultural traits in BC_2F_2 plants.

Figure S5. Grain appearance in BC_2F_2 plants.

Figure S6. Purity identification of hybrid rice seeds.

Figure S7. The average yield per mu among different hybrid combinations of GLY676 in Hainan and Shanghai.

Figure S8. Comparisons of agronomic traits in transgenic plants of R534.

Figure S9. Heading date in multiple hybrid combinations of GLY676 and QYSM.

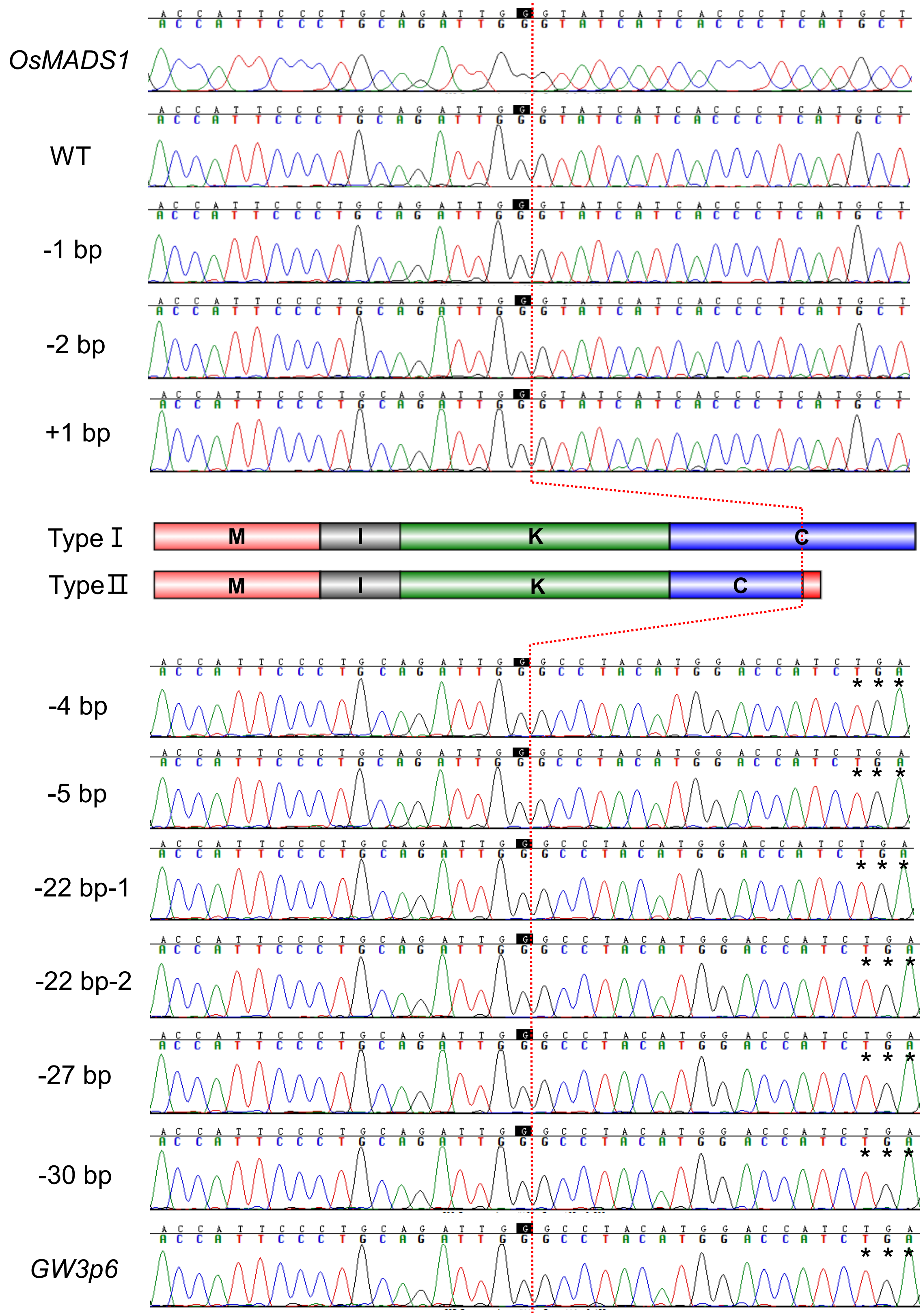

Figure S1 The electropherograms of cDNA sequencing in T_2 transgenic plants. The red dotted line represents the position in the junction of the seventh and eight exon of *OsMADS1*. The asterisks show the position of stop codon. The schematic illustration in the middle indicates the functional domains of OsMADS1 (upper) and $OsMADS1^{GW3p6}$ (lower).

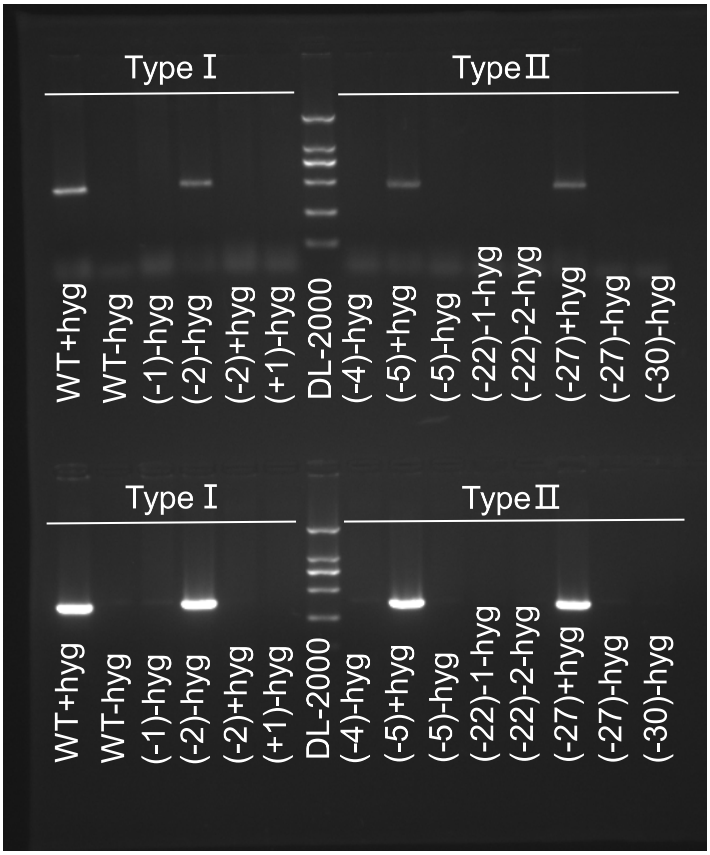

Figure S2 PCR amplicons of *Cas9* in BC_2F_2 and T_2 plants. All positive plants with *Cas9* gene are conducted in T_2 lines. The upper and lower parts indicate two genomic fragments of *Cas9*, respectively. The Arabic numerals in parentheses represent the number of inserted or deleted nucleotides.

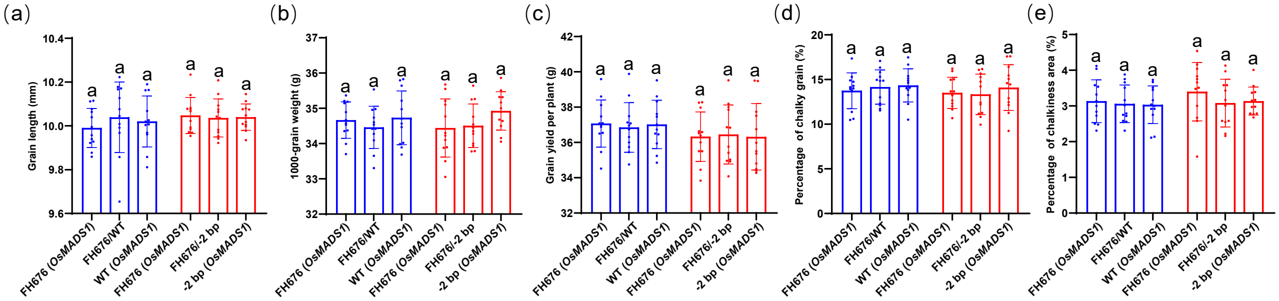

Figure S3 Statistical analyses of grain length (a), TGW (b), grain yield per plant (c), percentage of chalky grain (d), and percentage of chalkiness area (e) in BC_2F_2 lines of FH676 with Type I genotypes. The letters in parentheses represent the same mRNA as *OsMADS1*. Data shown as mean ± s.d. (n = 12 plants). Statistical analyses were performed by Duncan's multiple range tests.

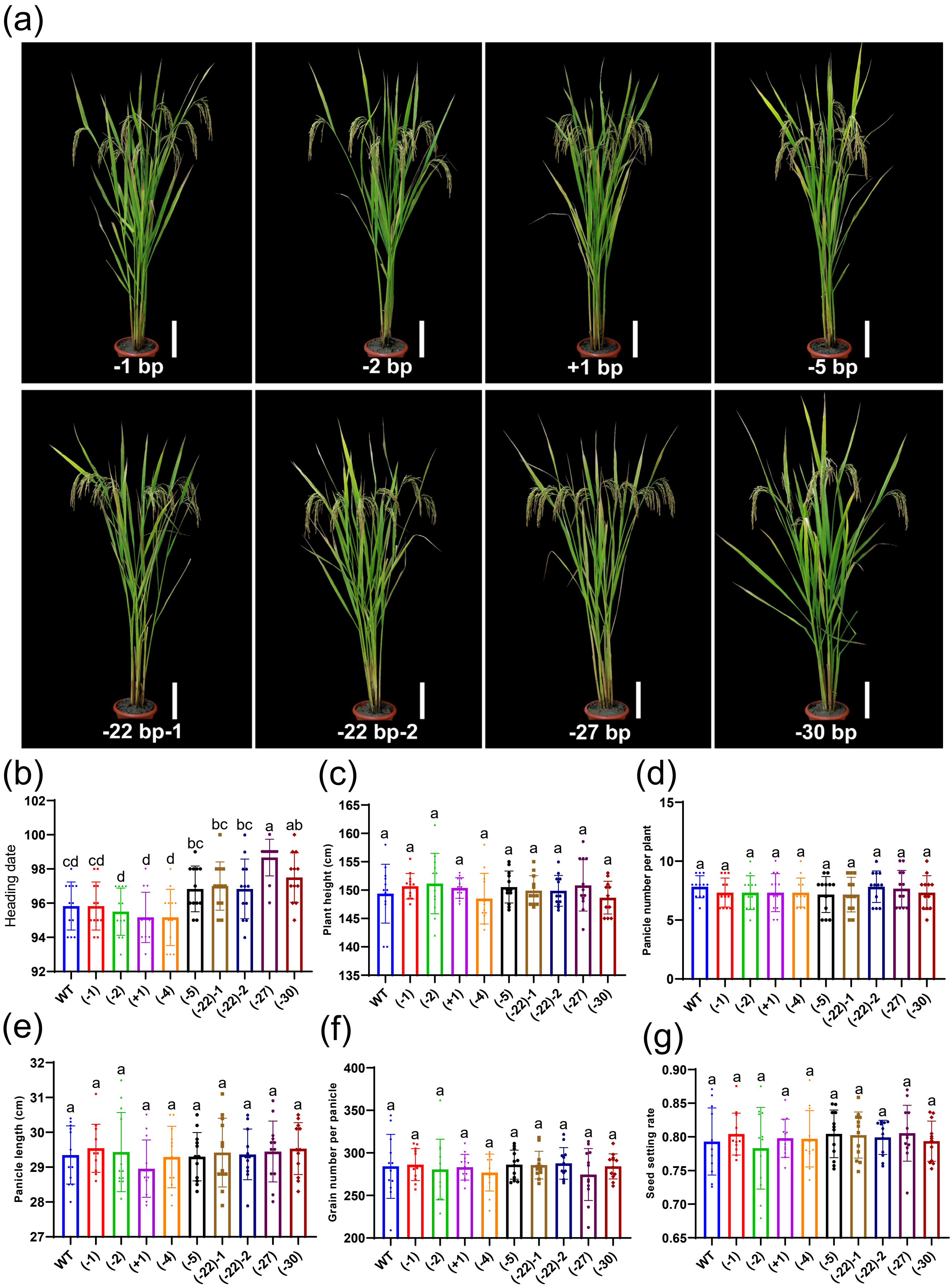

Figure S4 Comparisons of agricultural traits in BC_2F_2 plants. (a) Plant morphology in type I and type II plants. Scale bar = 10 cm. (b-g) Comparison of agricultural traits in type I and type II plants. (b) Heading date, (c) Plant height, (d) Panicle number per plant, (e) Panicle length, (f) Grain number per panicle, (g) Seed setting rate. The Arabic numerals in parentheses represent the number of inserted or deleted nucleotides. Values indicate means ± s.d. ($n = 12$ plants). Exact p values are performed by Duncan's multiple range test.

(a)

(b)

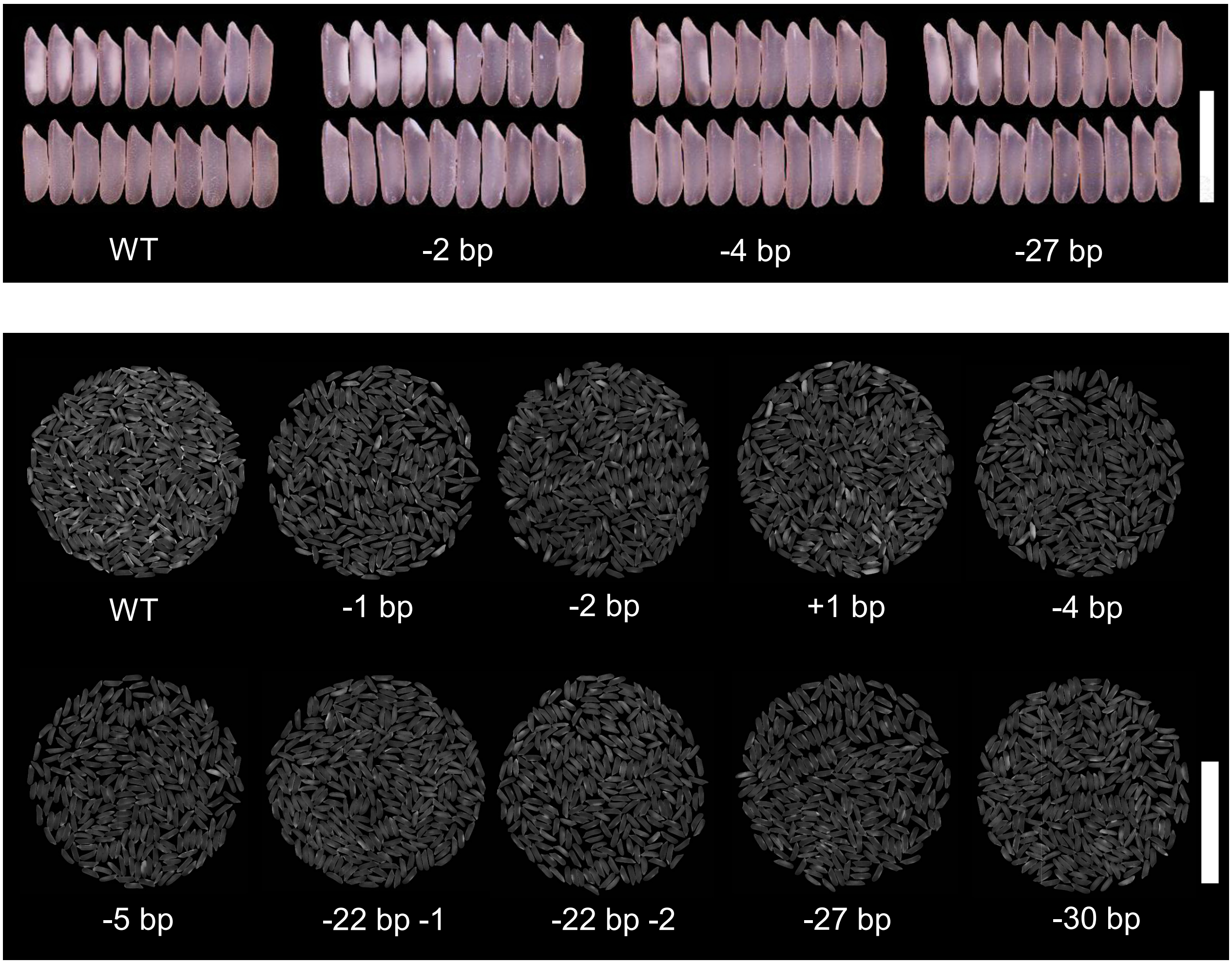

Figure S5 Grain appearance in BC_2F_2 plants. (a-b) Comparisons of grain endosperm transparency and chalkiness between Type I and Type II plants. (a) Scale bar=1 cm. (b) Scale bar=5 cm.

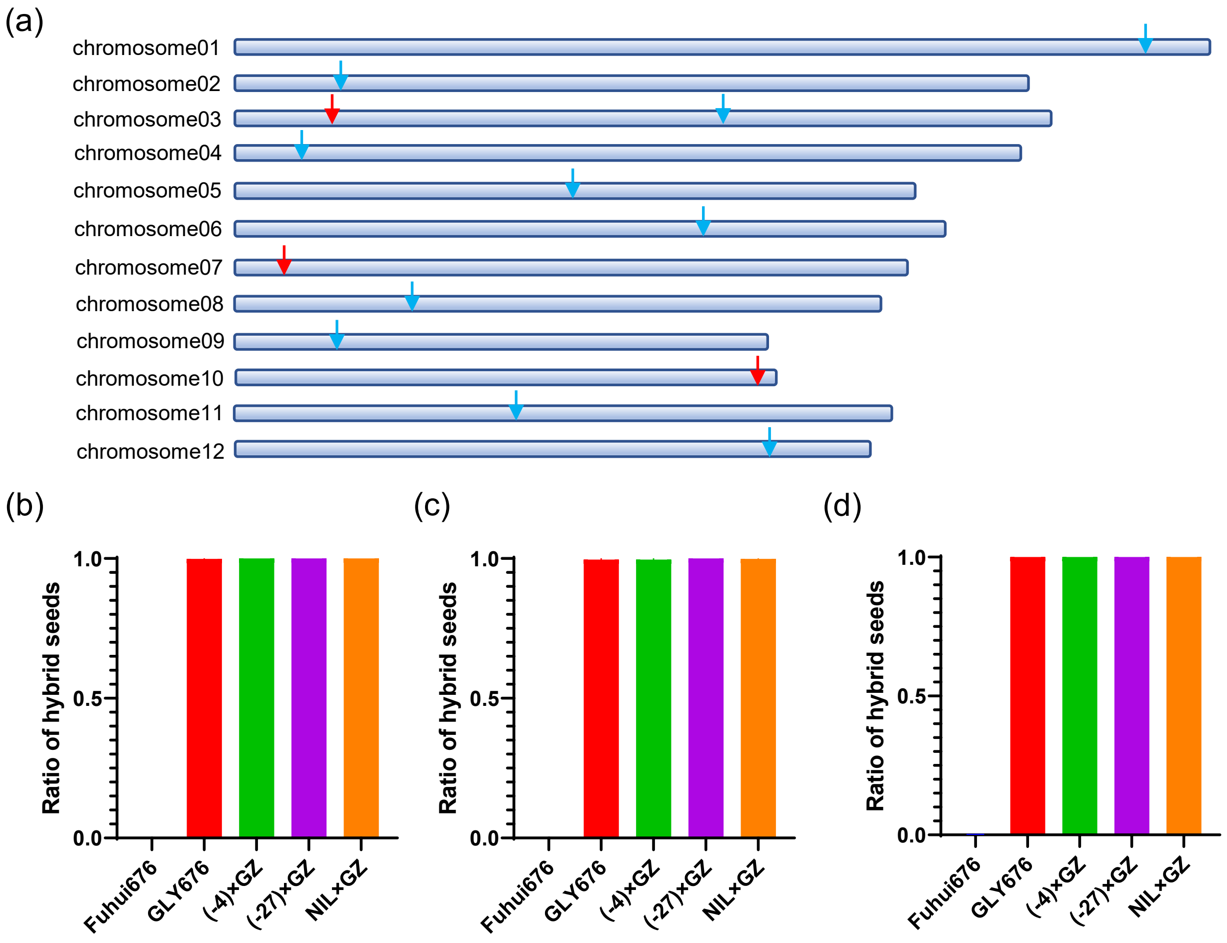

Figure S6 Purity identification of hybrid rice seeds. (a) Schematic illustration of the position of molecular markers on rice chromosomes. Red and blue arrows indicate InDel markers and SNP markers, respectively. (b-d) Ratio of hybrid seeds in (b) Hainan, 2020 (c) Shanghai, 2021 (d) Hainan, 2021. The Arabic numerals in parentheses represent the number of inserted or deleted nucleotides.

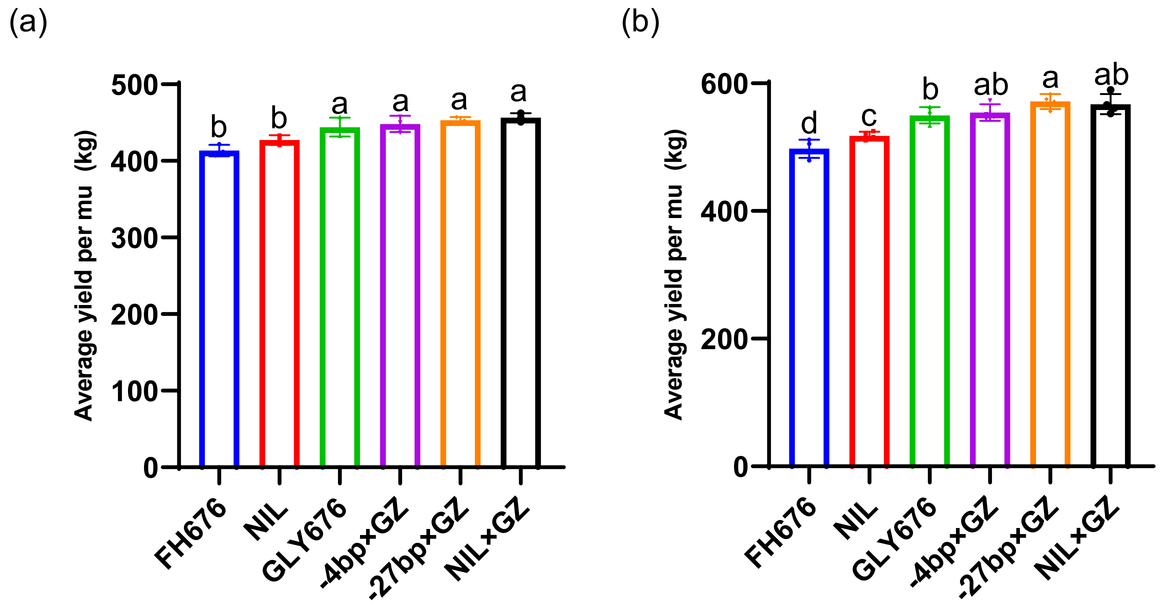

Figure S7 The average yield per mu among different hybrid combinations of GLY676 in Hainan province (a) and Shanghai (b). mu, 666.66 m^2 or a fifteenth hectare. Data shown as mean ± s.d. n = 3 paddies and 4 paddies in Hainan (a) and Shanghai (b). Statistical analyses were performed by Duncan's multiple range tests. The presence of the same lowercase letter denotes a non-significant difference between means ($p > 0.05$).

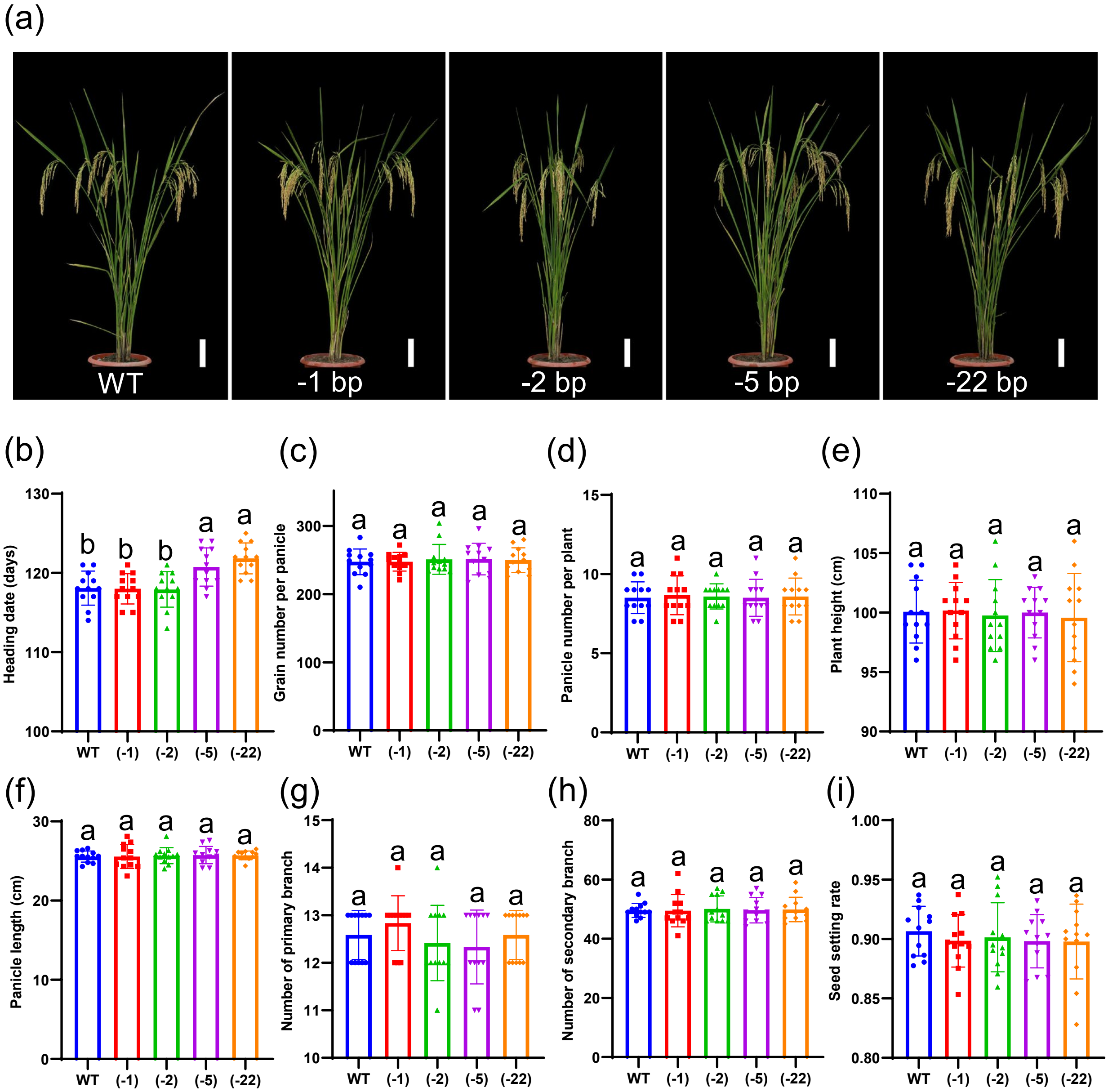

Figure S8. Comparisons of agronomic traits in transgenic plants of R534. (a) Plant morphology in type I and type II plants of R534. Scale bar = 10 cm. (b-i) Heading date (b), grain number per panicle (c), panicle number per plant (d), plant height (e), panicle length (f), number of primary branch (g), number of secondary branch (h), seed setting rate (i) of transgenic plants of R534. The Arabic numerals in parentheses represent the number of inserted or deleted nucleotides. Data shown as mean ± s.d. (n = 12 plants). Statistical analyses were performed by Duncan's multiple range tests. The presence of the same lowercase letter denotes a non-significant difference between means ($p > 0.05$).

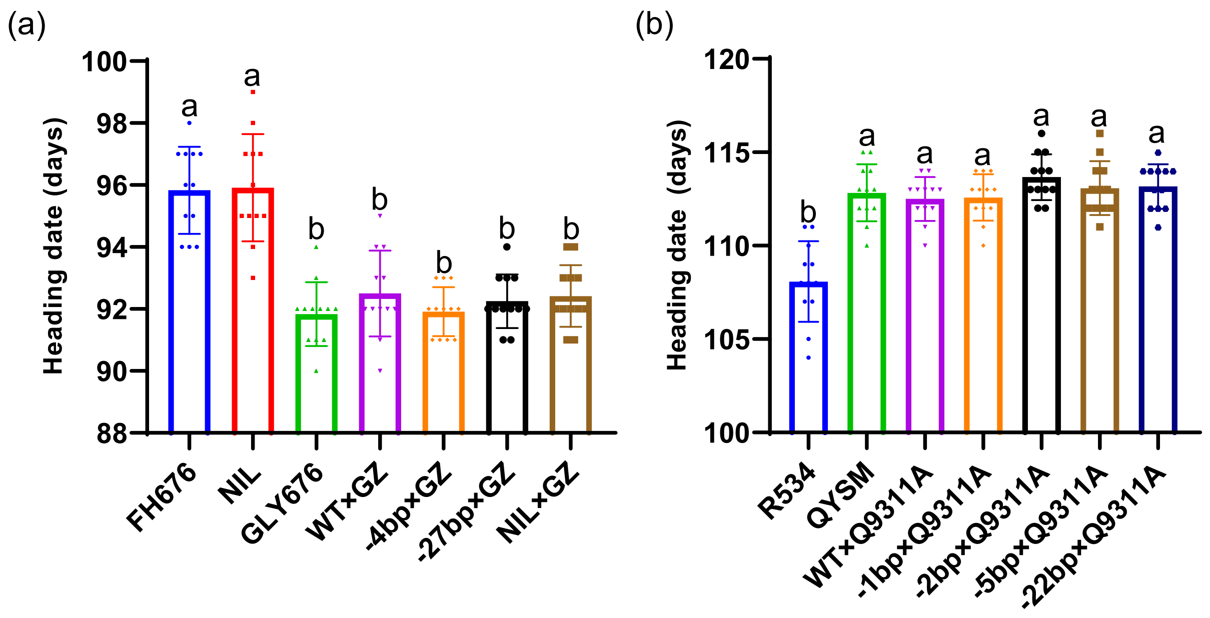

Figure S9 Heading date in multiple hybrid combinations of GLY676 (a) and QYSM (b). The heading date statistics for the hybrid combinations of GLY676 and QYSM were conducted in Hainan and Shanghai, respectively. Data shown as mean ± s.d. (n = 12 plants). Statistical analyses were performed by Duncan's multiple range tests. The presence of the same lowercase letter denotes a non-significant difference between means ($p > 0.05$).
