# Supplementary material for: A higher‐yield hybrid rice is achieved by assimilating a dominant heterotic gene in inbred parental lines
Source: Plant Biotechnol J. 2024 Mar 7;22(6):1669–80. doi: 10.1111/pbi.14295 (PMC11123404; doi:10.1111/pbi.14295)
Supplement: Supplementary file 5 — Table S1 Progress of the genetic basis of heterosis in plants. [file PBI-22-1669-s004.docx]

Table S1. Progress of the genetic basis of heterosis in plants

| Genetic Model | Genetic materials | Genetic populations | Species | Reference |
| --- | --- | --- | --- | --- |
| Dominance | 91 diallel-cross F_1_ hybrids and 12 founder inbred lines | F_1_ hybrids | Maize (Zea mays) | (Wang et al., 2023) |
| Dominance | 1,495 elite hybrid rice varieties | F_1_ hybrids | Rice (*Oryza sativa*) | (Huang et al., 2015) |
| Dominance | 2,839 rice hybrids and 9,839 F_2_ lines | F_1_ and F_2_ | Rice (*Oryza sativa*) | (Gu et al., 2023) |
| Dominance | 66 F_1_ hybrids and 12 inbred parents | F_1_ hybrids | Maize (Zea mays) | (Yang et al., 2017) |
| Epistasis | 1604 hybrids and their 135 parental elite breeding lines | F_1_ hybrids | Bread wheat (*Triticum aestivum*) | (Jiang et al., 2017) |
| Dominance | 435 individuals derived from intercrosses of 30 parents | F_1_ hybrids | *Arabidopsis thaliana* | (Seymour et al., 2016) |
| Dominance | 10074 F_2_ lines from 17 representative hybrid rice crosses | F_2_ | Rice (*Oryza sativa*) | (Huang et al., 2016) |
| Dominance,  Overdominance | 5360 offsprings from three elite maize hybrids | F_2_ | Maize (Zea mays) | (Liu et al., 2020) |
| Dominance,  Overdominance | 184 individuals in F_2_ and F_2:3_ populations separately | F_2_ and F_2:3_ | Oilseed rape | (Ye et al., 2023) |
| Epistasis | 250 F_2:3_ families | F_2:3_ | Rice (*Oryza sativa*) | (Yu et al., 1997) |
| Dominance,  Overdominance,  Epistasis | 173 F_2:3_ family lines | F_2:3_ | Upland cotton (Gossypium hirsutum L.) | (Liang et al., 2015) |
| Dominance,  Overdominance,  Epistasis | 250 DH lines and their 250 corresponding testcross hybrids | DH and TC | Rapeseed (*Brassica napus L.*) | (Radoev et al., 2008) |
| Overdominance | 76 ILs | IL and TC | Tomato (*Solanum lycopersicum*) | (Semel et al., 2006) |
| Epistasis | 1400 BILs | BIL | Tomato (*Solanum lycopersicum*) | (Torgeman and Zamir, 2023) |
| Dominance | 388 BC_I_F_7_ lines, 194 F_8_ lines, two parents, F_1_ | RIL and BC | Rice (*Oryza sativa*) | (Xiao et al., 1995) |
| Pseudo-overdominance | 1253 hybrids derived from crosses between parents and RILs | RIL and TC | Maize (Zea mays) | (Larièpe et al., 2012) |
| Pseudo-overdominance | 250 RILs | RIL | Sorghum (*Sorghum Moench*) | (Li et al., 2015) |
| Dominance | 219 RIL lines and a backcross population | RIL and RILBC_1_ | Rice (*Oryza sativa*) | (Li et al., 2016) |
| Dominance,  Overdominance,  Epistasis | 194 F_7_ lines, 222 F_12_ lines and their backcross populations | RIL and BC | Rice (*Oryza sativa*) | (Li et al., 2008) |
| Additive and over- dominant effects, Epistasis | 281 BCF_1_ lines, 148 RILs | RIL and BC | Rice (*Oryza sativa*) | (Luo et al., 2009) |
| Overdominance,  Epistasis | 254 F_10_ RILs, 2 BC_1_F_1_ and 2 testcross populations | RIL, BC and TC | Rice (*Oryza sativa*) | (Li et al., 2001; Luo et al., 2001; Mei et al., 2003) |
| Dominance,  Overdominance,  Epistasis | 4 populations derived from 180 RILs | RIL and BC | Upland cotton (Gossypium hirsutum L.) | (Shang et al., 2016) |
| Dominance | 142 RILs and 3 testcrosses | RIL and TC | Maize (Zea mays) | (Frascaroli et al., 2007) |
| Overdominance,  Dominance,  Epistasis | 162 lines of CSILs and corresponding testcrosses | CSIL and TC | Upland cotton (Gossypium hirsutum L.) | (Guo et al., 2013) |
| Dominance,  Epistasis | 202 CSSLs | CSSL | Rice (*Oryza sativa*) | (Shen et al., 2014) |
| Dominance,  Overdominance,  Epistasis | 360 crossed generated by 240 RIL lines | IMF_2_ | Rice (*Oryza sativa*) | (Hua et al., 2003; Zhou et al., 2012) |
| Dominance, Overdominance,  Epistasis | 147 crosses generated by 294 RIL lines | IMF_2_ | Maize (Zea mays) | (Guo et al., 2014; Tang et al., 2010) |

DH, doubled haploid. TC, testcross. IL, introgression line. BIL, backcross inbred line. RIL, recombinant inbred lines. CSIL, chromosome segment introgression lines. CSSL, chromosome segment substitution lines. IMF_2_, immortalized F_2_ population.

Reference

Frascaroli, E., Canè, M.A., Landi, P., Pea, G., Gianfranceschi, L., Villa, M., Morgante, M., and Pè, M.E. (2007). Classical Genetic and Quantitative Trait Loci Analyses of Heterosis in a Maize Hybrid Between Two Elite Inbred Lines. Genetics *176*, 625-644.

Gu, Z., Gong, J., Zhu, Z., Li, Z., Feng, Q., Wang, C., Zhao, Y., Zhan, Q., Zhou, C., Wang, A.*, et al.* (2023). Structure and function of rice hybrid genomes reveal genetic basis and optimal performance of heterosis. Nat Genet *55*, 1745-1756.

Guo, T., Yang, N., Tong, H., Pan, Q., Yang, X., Tang, J., Wang, J., Li, J., and Yan, J. (2014). Genetic basis of grain yield heterosis in an “immortalized F_2_” maize population. Theor Appl Genet *127*, 2149-2158.

Guo, X., Guo, Y., Ma, J., Wang, F., Sun, M., Gui, L., Zhou, J., Song, X., Sun, X., and Zhang, T. (2013). Mapping Heterotic Loci for Yield and Agronomic Traits Using Chromosome Segment Introgression Lines in Cotton. J Integr Plant Biol *55*, 759-774.

Hua, J., Xing, Y., Wu, W., Xu, C., Sun, X., Yu, S., and Zhang, Q. (2003). Single-locus heterotic effects and dominance by dominance interactions can adequately explain the genetic basis of heterosis in an elite rice hybrid. P Natl Acad Sci USA *100*, 2574-2579.

Huang, X., Yang, S., Gong, J., Zhao, Q., Feng, Q., Zhan, Q., Zhao, Y., Li, W., Cheng, B., Xia, J.*, et al.* (2016). Genomic architecture of heterosis for yield traits in rice. Nature *537*, 629-633.

Huang, X., Yang, S., Gong, J., Zhao, Y., Feng, Q., Gong, H., Li, W., Zhan, Q., Cheng, B., Xia, J.*, et al.* (2015). Genomic analysis of hybrid rice varieties reveals numerous superior alleles that contribute to heterosis. Nat Commun *6*, 6258.

Jiang, Y., Schmidt, R.H., Zhao, Y., and Reif, J.C. (2017). A quantitative genetic framework highlights the role of epistatic effects for grain-yield heterosis in bread wheat. Nat Genet *49*, 1741-1746.

Larièpe, A., Mangin, B., Jasson, S., Combes, V., Dumas, F., Jamin, P., Lariagon, C., Jolivot, D., Madur, D., Fiévet, J.*, et al.* (2012). The Genetic Basis of Heterosis: Multiparental Quantitative Trait Loci Mapping Reveals Contrasted Levels of Apparent Overdominance Among Traits of Agronomical Interest in Maize (Zea mays L.). Genetics *190*, 795-811.

Li, D., Huang, Z., Song, S., Xin, Y., Mao, D., Lv, Q., Zhou, M., Tian, D., Tang, M., Wu, Q.*, et al.* (2016). Integrated analysis of phenome, genome, and transcriptome of hybrid rice uncovered multiple heterosis-related loci for yield increase. P Natl Acad Sci USA *113*, E6026-E6035.

Li, L., Lu, K., Chen, Z., Mu, T., Hu, Z., and Li, X. (2008). Dominance, Overdominance and Epistasis Condition the Heterosis in Two Heterotic Rice Hybrids. Genetics *180*, 1725-1742.

Li, X., Li, X., Fridman, E., Tesso, T.T., and Yu, J. (2015). Dissecting repulsion linkage in the dwarfing gene *Dw3* region for sorghum plant height provides insights into heterosis. P Natl Acad Sci USA *112*, 11823-11828.

Li, Z.-K., Luo, L.J., Mei, H.W., Wang, D.L., Shu, Q.Y., Tabien, R., Zhong, D.B., Ying, C.S., Stansel, J.W., Khush, G.S.*, et al.* (2001). Overdominant Epistatic Loci Are the Primary Genetic Basis of Inbreeding Depression and Heterosis in Rice. I. Biomass and Grain Yield. Genetics *158*, 1737-1753.

Liang, Q., Shang, L., Wang, Y., and Hua, J. (2015). Partial Dominance, Overdominance and Epistasis as the Genetic Basis of Heterosis in Upland Cotton (Gossypium hirsutum L.). PLOS ONE *10*, e0143548.

Liu, H., Wang, Q., Chen, M., Ding, Y., Yang, X., Liu, J., Li, X., Zhou, C., Tian, Q., Lu, Y.*, et al.* (2020). Genome-wide identification and analysis of heterotic loci in three maize hybrids. Plant Biotechnol J *18*, 185-194.

Luo, L.J., Li, Z.-K., Mei, H.W., Shu, Q.Y., Tabien, R., Zhong, D.B., Ying, C.S., Stansel, J.W., Khush, G.S., and Paterson, A.H. (2001). Overdominant Epistatic Loci Are the Primary Genetic Basis of Inbreeding Depression and Heterosis in Rice. II. Grain Yield Components. Genetics *158*, 1755-1771.

Luo, X., Fu, Y., Zhang, P., Wu, S., Tian, F., Liu, J., Zhu, Z., Yang, J., and Sun, C. (2009). Additive and Over-dominant Effects Resulting from Epistatic Loci Are the Primary Genetic Basis of Heterosis in Rice. J Integr Plant Biol *51*, 393-408.

Mei, H.W., Luo, L.J., Ying, C.S., Wang, Y.P., Yu, X.Q., Guo, L.B., Paterson, A.H., and Li, Z.K. (2003). Gene actions of QTLs affecting several agronomic traits resolved in a recombinant inbred rice population and two testcross populations. Theor Appl Genet *107*, 89-101.

Radoev, M., Becker, H.C., and Ecke, W. (2008). Genetic Analysis of Heterosis for Yield and Yield Components in Rapeseed (Brassica napus L.) by Quantitative Trait Locus Mapping. Genetics *179*, 1547-1558.

Semel, Y., Nissenbaum, J., Menda, N., Zinder, M., Krieger, U., Issman, N., Pleban, T., Lippman, Z., Gur, A., and Zamir, D. (2006). Overdominant quantitative trait loci for yield and fitness in tomato. P Natl Acad Sci USA *103*, 12981-12986.

Seymour, D.K., Chae, E., Grimm, D.G., Martín Pizarro, C., Habring-Müller, A., Vasseur, F., Rakitsch, B., Borgwardt, K.M., Koenig, D., and Weigel, D. (2016). Genetic architecture of nonadditive inheritance in Arabidopsis thaliana hybrids. P Natl Acad Sci USA *113*, E7317-E7326.

Shang, L., Wang, Y., Cai, S., Ma, L., Liu, F., Chen, Z., Su, Y., Wang, K., and Hua, J. (2016). Genetic analysis of Upland cotton dynamic heterosis for boll number per plant at multiple developmental stages. Sci Rep *6*, 35515.

Shen, G., Zhan, W., Chen, H., and Xing, Y. (2014). Dominance and epistasis are the main contributors to heterosis for plant height in rice. Plant Science *215-216*, 11-18.

Tang, J., Yan, J., Ma, X., Teng, W., Wu, W., Dai, J., Dhillon, B.S., Melchinger, A.E., and Li, J. (2010). Dissection of the genetic basis of heterosis in an elite maize hybrid by QTL mapping in an immortalized F_2_ population. Theor Appl Genet *120*, 333-340.

Torgeman, S., and Zamir, D. (2023). Epistatic QTLs for yield heterosis in tomato. Proc Natl Acad Sci U S A *120*, e2205787119.

Wang, B., Hou, M., Shi, J., Ku, L., Song, W., Li, C., Ning, Q., Li, X., Li, C., Zhao, B.*, et al.* (2023). De novo genome assembly and analyses of 12 founder inbred lines provide insights into maize heterosis. Nat Genet *55*, 312-323.

Xiao, J., Li, J., Yuan, L., and Tanksley, S.D. (1995). Dominance is the major genetic basis of heterosis in rice as revealed by QTL analysis using molecular markers. Genetics *140*, 745-754.

Yang, J., Mezmouk, S., Baumgarten, A., Buckler, E.S., Guill, K.E., McMullen, M.D., Mumm, R.H., and Ross-Ibarra, J. (2017). Incomplete dominance of deleterious alleles contributes substantially to trait variation and heterosis in maize. PLoS Genet *13*, e1007019.

Ye, J., Liang, H., Zhao, X., Li, N., Song, D., Zhan, J., Liu, J., Wang, X., Tu, J., Varshney, R.K.*, et al.* (2023). A systematic dissection in oilseed rape provides insights into the genetic architecture and molecular mechanism of yield heterosis. Plant Biotechnol J *21*, 1479-1495.

Yu, S.B., Li, J.X., Xu, C.G., Tan, Y.F., Gao, Y.J., Li, X.H., Zhang, Q., and Maroof, M.A.S. (1997). Importance of epistasis as the genetic basis of heterosis in an elite rice hybrid. P Natl Acad Sci USA *94*, 9226-9231.

Zhou, G., Chen, Y., Yao, W., Zhang, C., Xie, W., Hua, J., Xing, Y., Xiao, J., and Zhang, Q. (2012). Genetic composition of yield heterosis in an elite rice hybrid. Proc Natl Acad Sci U S A *109*, 15847-15852.
