# Supplementary material for: A higher‐yield hybrid rice is achieved by assimilating a dominant heterotic gene in inbred parental lines
Source: Plant Biotechnol J. 2024 Mar 7;22(6):1669–80. doi: 10.1111/pbi.14295 (PMC11123404; doi:10.1111/pbi.14295)
Supplement: Supplementary file 6 — Table S2 QTL mapping results in GLY676 F2 population. [file PBI-22-1669-s003.docx]

Table S2. QTL mapping results in GLY676 F_2_ population.

| Traits | Chr | Position | LOD | d/a | Average (FH) | Average (H) | Average (GZ) | Candidate gene |
| --- | --- | --- | --- | --- | --- | --- | --- | --- |
| Heading date | 1 | 40.25-40.95 | 5.289059 | -0.336050596 | 94.4008 | 95.5719 | 96.9141 | *MADS51* |
| Heading date | 2 | 24.05-24.55 | 3.978222 | 0.152137126 | 94.1047 | 95.8365 | 96.8866 | *OsCOL4* |
| Heading date | 3 | 3.25-3.45 | 4.105235 | 0.058910591 | 94.6679 | 95.5 | 97.1345 |  |
| Heading date | 8 | 4.25-4.55 | 143.97908 | -0.071749363 | 99.5875 | 97.439 | 87.8604 | *Ghd8* |
| Heading date | 10 | 17.05-17.35 | 5.222438 | -0.080838943 | 96.6264 | 95.6223 | 94.5252 | *Ehd1* |
| Heading date | 11 | 2.35-2.55 | 7.05002 | -0.292330988 | 96.2814 | 95.8102 | 94.6702 | *RCN1* |
| Plant height | 1 | 38.15-38.75 | 5.631027 | 0.1467663 | 129.35 | 125.091 | 123.254 | *sd1* |
| Plant height | 2 | 6.35-6.55 | 86.727493 | -0.658226588 | 129.39 | 130.499 | 110.029 | *tms5* |
| Plant height | 5 | 25.45-25.85 | 6.723631 | 0.39381575 | 129.468 | 125.162 | 122.823 | *SBI/OsGA2ox4* |
| Plant height | 8 | 4.15-4.45 | 30.944895 | -0.65415603 | 128.923 | 129.009 | 114.992 | *Ghd8* |
| Panicle length | 1 | 40.25-40.55 | 8.886834 | -0.004867758 | 28.7362 | 27.8446 | 27.0442 | *MADS51* |
| Panicle length | 8 | 2.5-2.95 | 4.913774 | -0.389124178 | 27.2937 | 27.724 | 28.6579 |  |
| Leaf length | 8 | 5.75-5.85 | 3.529363 | 3.545607882 | 41.2377 | 39.3091 | 39.408 |  |
| Leaf length | 9 | 21.35-21.75 | 5.144951 | -0.75274067 | 40.8331 | 40.4033 | 38.5122 |  |
| Leaf length | 11 | 1.55-1.85 | 3.879238 | -0.1452279 | 40.7236 | 39.916 | 38.639 |  |
| Leaf width | 1 | 5.15-5.35 | 31.266156 | 0.019002856 | 2.09266 | 1.98795 | 1.89355 | *D2/CYP90D2/SMG11* |
| Leaf width | 1 | 39.85-40.55 | 6.024698 | -0.33592812 | 1.96141 | 1.98488 | 2.0239 | *MADS51* |
| Leaf width | 7 | 24.35-24.75 | 5.725367 | -0.271546021 | 2.022 | 1.99585 | 1.94173 | *GE/CYP78A13/BG2* |
| Leaf width | 8 | 3.65-4.35 | 6.795514 | -0.605278266 | 2.0083 | 2.00606 | 1.9305 | *Ghd8* |
| Leaf width | 11 | 2.45-2.75 | 7.589119 | 0.014907508 | 2.0371 | 1.98651 | 1.94851 | *RCN1* |
| Leaf width | 12 | 2.75-3.45 | 4.428014 | 1.166324127 | 2.04421 | 1.98139 | 1.96256 |  |
| Tiller angle | 1 | 6-95-7.5 | 4.067204 | 0.590503817 | 71.1541 | 73.0736 | 71.3867 |  |
| Tiller angle | 2 | 6.05-6.55 | 6.316391 | -1.082017304 | 72.4885 | 74.2731 | 71.1655 | *tms5* |
| Tiller number | 1 | 5.85-6.65 | 5.684778 | 0.813292465 | 8.65314 | 8.96099 | 9.14338 |  |
| Tiller number | 3 | 22.05-23.05 | 15.82453 | 1.166875996 | 7.79853 | 9.2226 | 9.69038 |  |
| Tiller number | 7 | 27.55-28.35 | 4.16991 | -0.216024353 | 9.42231 | 9.04991 | 8.39194 |  |
| Tiller number | 8 | 4.25-4.55 | 4.639903 | -0.586727963 | 9.3253 | 9.17588 | 8.16216 |  |
| Fertility | 2 | 6.35-6.55 | 144.262697 | -0.619055895 | 0.966245 | 0.916949 | 0.274436 | *tms5* |
| Panicle number | 1 | 5.25-5.45 | 11.964489 | 0.984208011 | 7.01556 | 8.52347 | 8.75532 |  |
| Panicle number | 2 | 6.35-6.55 | 18.412222 | -0.438353812 | 7.40851 | 7.9263 | 9.659 | *tms5* |
| Panicle number | 3 | 22.05-23.05 | 9.015096 | 1.123689354 | 7.28986 | 8.46655 | 8.74359 |  |
| Panicle number | 6 | 28.5-29.55 | 4.662749 | -0.326310358 | 7.85621 | 8.12174 | 9.28889 |  |
| Panicle number | 7 | 28.35-29.45 | 5.17789 | 0.112369488 | 8.80738 | 8.27152 | 7.54694 |  |
| Panicle number | 8 | 4.15-4.45 | 5.069209 | -0.262844 | 7.97071 | 8.03697 | 8.90734 | *Ghd8* |
| Grain number per plant | 1 | 5.05-5.35 | 43.652645 | 0.463834278 | 302.888 | 261.269 | 243.688 |  |
| Grain number per plant | 2 | 6.25-6.55 | 6.601243 | -0.734939035 | 215.061 | 214.008 | 199.137 | *tms5* |
| Grain number per plant | 3 | 21.65-22.25 | 4.814621 | 2.602280896 | 255.007 | 270.497 | 270.335 |  |
| Grain number per plant | 9 | 14.15-14.25 | 3.824799 | 0.088010618 | 278.061 | 266.615 | 262.476 |  |
| seed setting rate | 2 | 6.35-6.55 | 91.074505 | -0.50879357 | 0.689543 | 0.666506 | 0.200259 | *tms5* |
| Grain weight | 1 | 6.85-7.65 | 7.843497 | -0.111865096 | 30.601 | 30.1367 | 29.6369 |  |
| Grain weight | 1 | 37.75-35.15 | 14.367432 | 0.354274663 | 30.7342 | 30.0807 | 29.6963 |  |
| Grain weight | 2 | 6.35-6.55 | 29.963575 | -0.671018074 | 30.311 | 30.5539 | 28.882 | *tms5* |
| Grain weight | 2 | 25.65-25.85 | 7.123024 | 0.77666246 | 29.62 | 30.427 | 30.2542 |  |
| Grain weight | 3 | 6.05-6.35 | 24.867617 | 0.229797117 | 29.1434 | 30.1801 | 31.0612 | *LGY3/OsMADS1/*  *GW3p6/OsLG3b* |
| Grain weight | 3 | 27.75-28.05 | 5.805345 | -0.165483292 | 30.4137 | 30.1735 | 29.6844 |  |
| Grain weight | 4 | 33.85-34.15 | 5.277024 | 4.305690927 | 29.5828 | 30.2877 | 30.3038 |  |
| Grain weight | 6 | 24.75-25.15 | 26.904733 | -0.190276315 | 30.8506 | 30.254 | 28.7568 | *TGW6* |
| Grain weight | 7 | 24.65-25.25 | 12.221702 | 0.459766651 | 29.3091 | 30.2667 | 30.565 | *GE/CYP78A13/BG2* |
| Grain weight | 8 | 3.65-4.25 | 28.677063 | -0.476763871 | 30.6232 | 30.3762 | 29.0998 |  |
| Grain weight | 9 | 20.95-21.45 | 5.653539 | -0.822547068 | 30.0743 | 30.3687 | 29.7937 |  |
| Grain weight | 11 | 2.65-3.45 | 14.701239 | -0.145872706 | 30.7993 | 29.3672 | 30.2051 |  |
| Grain weight | 12 | 26.65-26.95 | 7.503031 | 0.260521549 | 30.5204 | 30.0999 | 29.7126 |  |
| Grain yield per plant | 2 | 6.35-6.55 | 128.059488 | -0.692466535 | 30.9916 | 32.1648 | 7.50381 | *tms5* |
| Grain yield per plant | 3 | 22.05-23.05 | 5.673291 | 11.1351929 | 21.8445 | 27.5533 | 26.0813 |  |
| Grain yield per plant | 3 | 27.15-27.35 | 4.163189 | -2.317808621 | 23.1121 | 27.729 | 25.3143 |  |
| Grain yield per plant | 8 | 4.15-4.45 | 12.465668 | -0.671334265 | 26.8 | 27.8312 | 21.0665 |  |
| Grain yield per plant | 12 | 23.85-24.15 | 5.406321 | -0.671521806 | 27.8371 | 27.2882 | 21.99 |  |
| Grain length | 1 | 0.75-1.35 | 5.629635 | -0.092455621 | 10.279 | 10.222 | 10.1723 |  |
| Grain length | 1 | 34.65-35.55 | 25.002226 | 0.936164419 | 10.3172 | 10.208 | 10.157 |  |
| Grain length | 2 | 6.35-6.55 | 21.883169 | -0.424055452 | 10.2661 | 10.2502 | 10.0851 | *tms5* |
| Grain length | 2 | 33.85-34.75 | 10.154348 | 0.072981557 | 10.1623 | 10.226 | 10.2747 |  |
| Grain length | 3 | 5.95-6.15 | 46.252215 | 0.036875724 | 10.096 | 10.2267 | 10.3572 | *LGY3/OsMADS1/*  *GW3p6/OsLG3b* |
| Grain length | 3 | 24.95-25.25 | 6.088048 | 0.356858869 | 10.1608 | 10.2284 | 10.2712 | *GL3.1/qGL3* |
| Grain length | 6 | 21.45-21.75 | 16.471277 | 0.116497785 | 10.3187 | 10.2185 | 10.1323 |  |
| Grain length | 9 | 7.05-7.55 | 7.15608 | -0.540492324 | 10.2671 | 10.2352 | 10.1543 |  |
| Grain length | 10 | 11.95-12.55 | 5.911244 | -0.265608653 | 10.2766 | 10.2241 | 10.1466 |  |
| Grain length | 12 | 14-14.75 | 9.087808 | -0.04990657 | 10.1663 | 10.2238 | 10.266 |  |

Chr indicates the chromosome. The degree of dominance was quantified by calculating the ratio of *d*/*a*.
